# Cell Type Populations for 3D Anatomical Structures of the Human Reference Atlas

**DOI:** 10.1038/s41597-026-06642-4

**Published:** 2026-03-19

**Authors:** Andreas Bueckle, Bruce W. Herr, Lu Chen, Daniel Bolin, Danial Qaurooni, Michael Ginda, Yashvardhan Jain, Aleix Puig-Barbe, Kristin Ardlie, Fusheng Wang, Katy Börner

**Affiliations:** 1 Department of Intelligent Systems Engineering, Luddy School of Informatics, Computing, and Engineering, Indiana University, Bloomington, IN 47408 USA; 2 Department of Computer Science, Stony Brook University, Stony Brook, NY 11794 USA; 3 European Molecular Biology Laboratory-European Bioinformatics Institute, Wellcome Genome Campus, Hinxton, Cambridge CB10 1SD UK; 4 Broad Institute, Cambridge, MA 02142 USA; 5 Department of Biomedical Informatics, Stony Brook University, Stony Brook, NY 11794 USA

**Keywords:** Software, Data integration, Data processing, Data publication and archiving, Databases

## Abstract

The human body contains ~27–37 trillion cells of up to 10,000 cell types (CTs) within a volume of ~62–120 liters (males) and 52–89 liters (females). The Human Reference Atlas (HRA) v2.3 provides a quantitative 3D framework of CTs across 73 reference organs and 1,283 3D anatomical structures (ASs). The HRA Cell Type Population (HRApop) effort has quantified CTs per AS using high-quality single-cell datasets processed through scalable, reproducible workflows and cell type annotation (CTann) tools. HRApop v1.0 includes reference CT populations for 73 ASs (112 when sex-specific) using 662 datasets spatially registered to 230 locations across 17 organs (31 when sex-specific). For 558 single-cell (sc-)transcriptomics datasets (11,042,750 cells), CTs and biomarker expressions were computed using Azimuth, CellTypist, and popV. To test generalizability, 104 sc-proteomics datasets (16,576,863 cells) were integrated. In total, HRApop includes 27,619,613 cells and serves as a healthy reference for researchers aiming to elucidate mechanisms underlying cellular interactions as well as cellular and tissue level disease progression, which may facilitate advancements in basic discovery and lead to new therapeutic strategies.

## Background & Summary

### The need

The volume^1^ of the adult human body is estimated to range from 62–120 liters (0.062–0.120 m³) in males with 36 or 37^2^^,^^3^ trillion cells to 52–89 liters (0.052–0.089 m³) in females with 28 trillion cells^3^. There is no consensus on the number of CTs within the human body. Estimates range from 400 major CTs^2–5^ to 3,358 total CTs^6^, and depend on the criteria used to determine what constitutes a CT (see the **Estimates of number of CTs in the human body** section for a more detailed discussion). Efforts like the Human BioMolecular Atlas Program (HuBMAP)^7,8^, the Human Cell Atlas^9–11^, and many of the 20+ other atlas efforts contributing to the HRA aim to provide clarity based on high-quality experimental data collection and analysis.

Most atlas projects attempt to capture the number and type of cells per AS together with biomarker expression values, based on expert knowledge or experimental data. For example, the Blue Brain Cell Atlas^12^ (bbp.epfl.ch/nexus/cell-atlas) features 3D data for the mouse brain with *CT populations* (see Box 1 for a definition of this term and all others that are written *in italics* when first mentioned), programmatically placed inside 737 brain regions defined in the Allen Mouse Brain Atlas^13^; different ASs, regions, and their CT populations can be toggled on and off, and the color hue can be set to encode cell regions, types, density, and other properties; cell counts for neurons and glia (with confidence values) are also displayed. The Genotype-Tissue Expression (GTEx) portal^14^ (gtexportal.org/home/singleCellOverviewPage) features CT populations from 25 tissue blocks in eight organs from 16 *donors*, and the Chan Zuckerberg Initiative CELLxGENE Portal^15^ (cellxgene.cziscience.com/datasets) features CT populations for 28 human organs but without visual representations of these structures. Visualization of functional tissue unit level^16^ relyon Anatomograms^17,18^, which combine 2D medical illustrations with associated CT populations, have been used acrossprojects, including the Gene Expression Team at the European Molecular Biology Laboratory-European BioinformaticsInstitute in collaboration with the Wellcome Sanger Institute; the Kidney Precision Medicine Project^19,20^ Explorer (atlas.kpmp.org/explorer); and the HRA Functional Tissue Unit Explorer (apps.humanatlas.io/ftu-explorer).

Given that life unfolds in 3D, there is a strong interest to capture CT populations for ASs in the human body in 3D. The HRA v2.3 features the 3D shape, size, location, and rotation of 1,283 3D ASs, which can be explored at humanatlas.io/3d-reference-library; each 3D AS belongs to one of 73 organs, also called 3D reference objects^21^. As new organ experts join the HRA effort, new 3D structures for male and female organs are added. Data from portals such as GTEx, CELLxGENE, KPMP (atlas.kpmp.org), the HuBMAP Data Portal (portal.hubmapconsortium.org)^22^, and the Cellular Senescence Network (SenNet) Data Portal^23,24^ (data.sennetconsortium.org/search) can be spatially registered into the 3D reference objects using the web-deployed HRA Registration User Interface (RUI, apps.humanatlas.io/rui)^25,26^, guided by standard operating procedures^27–29^.

### The challenge

Computation of CT populations for the many different ASs in the human body requires both a 3D registration and reproducible dissociation protocols that allow isolating single cells (or single nuclei) from tissue in support of sc-transcriptomics analyses^30–32^ or high-quality cell segmentation for sc-proteomics spatial data^33–35^. In both cases, CTann tools are needed to assign a CT to each cell. Most single-cell segmentation and annotation tools are organ-specific. However, human organs are large (an average human kidney is about 10–12 cm long, 5–7 cm wide, and 3 cm thick and is estimated to contain about 110 billion cells^3^). Organs have many different internal ASs with vastly different CT populations that serve diverse physiological functions. The HRA^36,37^ uses the Visible Human Project data^37–40^ and the expertise of medical illustrators to segment 1,283 3D ASs in 37 vital organs in the HRA v2.3. When given tissue data that is spatially registered into these 3D AS using the RUI^25^, as well as single-cell dissociation/segmentations and annotations^33,41–49^, the number of cells per CT can be computed for specific ASs, i.e., 15 AS within the male lung, see Fig. 3. To compute HRApop at the AS (not organ) level, 16,293 datasets from four different portals were downloaded, metadata for *donors* was harmonized, and one or multiple CTann tools were run for each sc-transcriptomics dataset. This paper explains how 662 high-quality datasets were chosen to compute dataset, extraction site, and AS-specific CT populations and biomarker expression values using scalable, reproducible workflows, how open HRApop v1.0 data was published, and what known limitations exist.

### The opportunity

The 27,619,613 cells in HRApop v1.0 represent CT populations for 73 ASs (112 when sex-specific) across 17 organs (31 when sex-specific). CTs and biomarker expressions were computed using Azimuth^50^, CellTypist^51,52^, and/or popV^53^ for sc-transcriptomics datasets. These CT populations at the AS level can serve as al healthy reference for stakeholders working on the processes behind cell-to-cell communication, functional tissue unit activity, and disease advancement in cells and tissues, which could enable progress in foundational research and the development of innovative therapies. To make the HRApop v1.0 dataset available for single-cell biologists, bioinformaticians, computational biologists, and physician scientists, it was published as 5-Star Linked Open Data^54^, with full provenance including donor metadata and resolvable Uniform Resource Identifiers, making it a Findable, Accessible, Interoperable, and Reusable (FAIR^55^) resource.

HRApop data products can be accessed in various HRA applications and the HRA Application Programming Interface (API, apps.humanatlas.io/api). Additionally, SPARQL (www.w3.org/TR/sparql11-query) queries can be run to, e.g., retrieve all AS-CT combinations, with sex, tool, CT, and cell percentage as CSV files (see **Data Records** section). Beyond direct download, the **Usage Notes** section points to Python example code for using the HRA API to get Anatomical Structure Cell Type Populations (*ASpop*), i.e., the number of cells per CT for an AS, as well as Dataset and Extraction Site Cell Type Populations (*DESpop*), i.e., the number of cells per CT for all datasets that meet quality *Criteria C1-4* (see **Methods**) and their extraction sites, via the HRA Knowledge Graph (KG)^21^. A companion website for this paper is available at cns-iu.github.io/hra-cell-type-populations-supporting-information.

## Overview

The HRApop effort combined (1) CT populations from sc-transcriptomics and sc-proteomics data made with CTann tools (or via user-assigned CT label), (2) donor metadata, and (3) 3D extraction sites recorded via the RUI. This involved running two scalable workflows, optimized to efficiently deal with the continuously growing number of datasets. Over the last three years, the processes used to Download data, perform Cell Type Annotations, and compute CT populations per dataset (called **DCTA Workflow**) and the workflow that takes CT populations, donor metadata, and 3D extraction sites via the RUI, to compute Cell Type Populations for ASs, datasets, and extraction sites (called **RUI2CTpop Workflow**) were implemented. Note that CT populations record the number of cells per CT not just for ASs but also for datasets and extraction sites.  The top-10 biomarkers per CT and their mean expression values were computed for datasets (see **Methods**).

The **Methods** section also details relevant terminology and describes implementation of the DCTA and RUI2CTpop Workflows. The **Technical Validation** section shows confidence scores per cell per tool, gene counts, the prevalence of different CTs inside not just the organs but also the ASs of the human body, and the number of datasets per organ and AS by sex and CTann tool.

## Generalization to spatial data

In HRApop v1.0, 104 sc-proteomics datasets contributed 16,576,863 cells, which brought the total number of cells to 27,619,613. They were associated with high-quality publications^36,56–60^ and used protein and antibody-based modalities such as cyclic Immunofluorescence^61^, Cell DIVE^62–64^, and co-detection by indexing^65^ to identify proteins and quantify their expressions in a tissue *in situ. *Adding iterative bleaching extends multiplexicity (IBEX)^66,67^ is planned. While this paper does not focus on sc-proteomics data, it presents a curated collection of sc-proteomics datasets as a generalized use case for HRApop. Spatial proteomics has received significant interest from the scientific community in recent years^68^, which has led to increased high-quality data generation. If integrated in HRApop, it enables the creation of CT populations with a preserved spatial context for each cell, which is lost in sc-transcriptomics datasets, although some recent work has attempted recoveries for specific assay types and organs^49^.

The DCTA Workflow has output CT populations and metadata for all datasets, both sc-transcriptomics and sc-proteomics. While sc-transcriptomics datasets were run through at least one CTann tool, sc-proteomics datasets had CTs assigned by human experts, see CT populations as cell tables linked in Table S1. The DCTA Workflow read a list of sc-proteomics datasets (available on GitHub^69^) and generated a CT population (JSON) as an input for the RUI2CTpop Workflow (see GitHub^70^). In the future, the DCTA Workflow will be extended to (1) handle other generalized use cases that cannot be annotated with CTann tools and (2) run more CTann tools over new and existing HRApop datasets for additional CT populations.

To align CT annotations with Cell Ontology (CL, www.ebi.ac.uk/ols4/ontologies/cl)^71–73^, CT labels were shared by contributors, see this related publication^35^ and lod.humanatlas.io/ctann/vccf/latest for crosswalks (see also **Methods** section). Unmapped CTs are on GitHub^74^.

## Estimates of number of CTs in the human body

There is no consensus on the number of CTs within the human body, but estimates range from 400 major CTs^2–5^ to 3,358 CTs^6^, depending on how a CT is defined. As an example, in the retina, a major class of retinal neurons is the amacrine cell (purl.obolibrary.org/obo/CL_0000561). This cell can be subdivided in multiple ways. At a broad level, it is often classified into GABAergic (purl.obolibrary.org/obo/CL_4030027) and glycinergic (purl.obolibrary.org/obo/CL_4030028) types (two categories). However, based on morphology, researchers have identified 25 distinct amacrine CTs^75^. At the level of sc-transcriptomics, the Human Retina Cell Atlas^76^ has reported 123 CTs in the retina, including 73 molecularly distinct amacrine CTs. As a result, depending on the resolution—functional class, morphology, or transcriptomic profile—one might count one, two, 25, or 73 different amacrine CTs. In the case of the human brain, sampling more than three million nuclei from approximately 100 dissections across the forebrain, midbrain, and hindbrain, 461 clusters and 3,313 subclusters (granular CTs) organized largely according to developmental origins were identified^77^. Combining CTs in the Human Lung Cell Atlas^78^ and the CellRef atlas from the LungMAP consortium^79^ revealed 68 distinct CTs in the human lung and nasal cavity^80^. In August 2025, CL^71^ contained 3,358 classes, but this included many non-human CT terms as well as grouping classes—i.e., internal nodes in the CT typology that do not correspond to distinct, terminal CTs. When limiting the count to leaf-level human CTs, the number was in the order of 2,500, but this did not reflect many of the novel CTs that have been recently defined using single-cell technologies. Further, about 2,000 CTs^6^ in CL^71–73^ are connected to ASs in the cross-species anatomy ontology Uberon^81,82^ via ‘part of’ relationships. As a result, 400 total CTs might be a clear underestimation. Assuming that there are 78 major organs in the human body of varying size and cellular complexity, with most organs averaging between 50 and 120 transcriptomic CTs, aside from the brain with 3,000–5,000, there might be close to 10,000 CTs in adult mammalian organisms, depending on the criteria for distinguishing CTs.

## Limitations

HRApop v1.0 comes with a number of limitations:

### Dataset duplication across data portals

Some datasets (e.g., by KPMP) are available via multiple data portals (e.g., HuBMAP and CELLxGENE). However, no dataset should be used twice for HRApop construction. Data duplication detection is difficult as different versions of the data might have different metadata. For example, there might be a dataset submitted with the very first paper submission that is linked to a preprint, a slightly expanded dataset associated with a revised and later preprint version, and a final version of the dataset linked to a peer-reviewed published paper; paper title and authors might also change in the process.

### CTann tools were trained on underspecified data

Azimuth, CellTypist, and popV were trained on high-quality reference datasets that might lack metadata on donor demographics (e.g., age, sex, body mass index/BMI, ethnicity), and for which RUI extraction sites were not available; i.e., tissue samples whose precise location within an organ is unknown, were used to train these CTann tools. HRApop, however, utilizes existing CTann tools to compute ASpop that are specific to the diverse ASs and tailored to the male and female human body. As more RUI registered sc-transcriptomics datasets become available, CTann tool developers might like to use this additional tissue origin information to optimize CTann training and annotation predictions.

### Missing assay type information needed for batch correction

At present, the ds-graph HRA Digital Object type^21^, which captures datasets and their extraction sites plus donor metadata, only contains assay type metadata as a free string rather than an ontology term (e.g., 0 × 3’ v1-3, 10x scATAC-seq, MERFISH, Smart-seq, all listed on the CELLxGENE portal). The HRA KG will be extended to provide look-up tables for assay types to ontology terms for enabling more systematic queries. Also, collaboration is ongoing with the HuBMAP and SenNet portal teams to utilize ontology terms for identifying and standardizing assay types.

### Intersecting 3D reference objects

The 1,283 3D ASs are supposed to have no overlap with each so that CT populations are specific to one, not multiple intersecting ASs. However, in HRA v2.3, 18 ASs in 17 organs (e.g., two in the female heart) have intersections with each other and also have extraction sites in them (labeled “TB3” in Fig. S1). The AS-AS pairs with the most (here three) extraction sites that collide with both ASs are the ‘outer cortex of kidney’ and the ‘renal pyramid A’ in the male, left kidney as well as the ‘kidney capsule’ and the ‘outer cortex of kidney’ in the female, left kidney. Intersections will be corrected and tissue blocks will be re-registered for an upcoming HRA release.

### Limited coverage of organs and ASs

HRApop aims to compute CT populations for all 1,283 3D ASs in 73 3D reference objects of the entire HRA v2.3. However, in v1.0, only 73 3D ASs (112 if male and female are counted separately) in 17 organs (31 if male and female are counted separately) had data to compute AS-specific CT populations. The **Technical Validation** section expands on current HRApop coverage of organs and ASs.

Plans for improving the computation, coverage, and quality of future HRApop releases are outlined in the Supplementary Information.

## Methods

### Terminology

Box 1 introduces key terminology for computing and using HRApop; it also provides links to concepts already defined in related papers. Terms defined in this Box are written* in italics* when first mentioned in this paper.

**Box 1** HRApop key terminology. Other HRA terms are defined in the HRA Glossary^136^

**Fig. 1:**
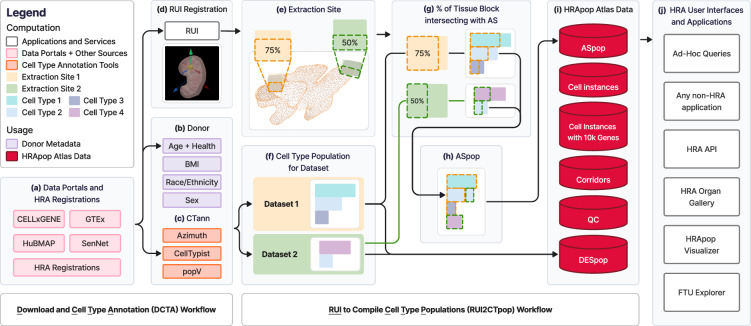
Computation and usage of HRApop. (**a**) Data is ingested from four data portals and/or from HRA Registrations^87^. **(b)** Donor information is extracted from dataset metadata or the extraction site (if from HRA Registrations). **(c)** CT populations are computed using CTann tools. **(a**–**c)** is handled by the **DCTA Workflow**. **(d)** RUI-assigned extraction sites for each dataset are identified. If a dataset has an extraction site, **(e)** *intersection* *percentages* of the extraction site with ASs can be computed. **(f)** CT populations for corresponding datasets obtained via one or more CTann tools can be combined with **(g)** intersection percentages between extraction site and AS, resulting in **(h)** ASpop, which, together with DESpop, are then published as **(i)** HRApop Atlas Data. **(d**–**i)** is handled by the **RUI2CTpop Workflow**. **(j)** illustrates usage of *HRApop Atlas Data* by applications inside and outside the HRA data ecosystem.

- **Anatomical Structure Cell Type Populations (ASpop)**: Captures the number of cells per CT for an *AS.* Metadata includes ontology ID, donor sex, *AS* label, the *CTann tool* used to assign the *CTs*, and a list of datasets from which the data was sourced. An exemplary *ASpop* is shown on the companion website at cns-iu.github.io/hra-cell-type-populations-supporting-information/#for-an-as.
- **Cell instance**: Is an occurrence of a unique cell, usually identified by its bar code or a unique string. In the data products of HRApop v1.0 (see **Data Records**), cells can be annotated by multiple CTann, which can lead to potentially multiple labels for the same cell.
- **Cell type (CT) population**: Is a listing of unique CTs and their counts computed for *ASs, extraction sites*, and datasets. For the latter, mean biomarker expression values are also computed. *CT populations* are computed from *CT* counts in *experimental datasets*, obtained either via *CTann* in the *DCTA Workflow* (for *sc-transcriptomics* datasets), or via expert/author-provided annotations (*sc-proteomics* datasets). *CT populations* are associated with exactly one *CTann* tool; if a dataset has *CT populations* from multiple *CTann* tools, the *CTann* tool that computed the CT population becomes part of its provenance. *CT populations* are stored in the *ASpop* graph (for AS) and the *DESpop* (for datasets and extraction sites).
- **Collision detection (bounding box)**: Is a computationally efficient but imprecise method for identifying intersections between two 3D objects in Euclidean space. For each 3D object, the bounding box is a cuboid shape that encapsulates all vertices and only this bounding box is used when computing collisions.
- **Collision detection (mesh-based)**: A more precise but computationally more expensive method for identifying the intersection between two 3D objects in Euclidean space based on mesh surfaces, resulting in *AS* tags for extraction sites. To enable mesh-based collision detection, *3D reference objects* need to be pre-processed. Details are provided in the **Methods** section.
- **Corridor**: Describes a 3D volume that encompasses all possible locations for an *extraction site* while maintaining its *intersection percentages* with any *ASs* based on *mesh-based collision detection*, see Fig. 1e.
- **Criteria C1-4**: Describes four conditions that a dataset must fulfill in order to be used for computing HRApop: (1) it has an extraction site in a 3D reference object; (2) it has a *CT population* via *CTann* or the *sc-proteomics* workflow; (3) it comes from a data portal with quality assurance/quality control or has an associated peer-reviewed publication; and (4) it is from a healthy adult human *donor*.
- **Dataset and Extraction Site Cell Type Populations (DESpop, formerly “Atlas-Enriched Dataset Graph” in a related HRA publication**^36^**)**: Is the CT population for a collection of datasets and their *extraction sites* that meet *Criteria C1-4*.. A snippet of the *DESpop* is shown on the companion website at cns-iu.github.io/hra-cell-type-populations-supporting-information/#for-a-dataset.
- **Download and Cell Type Annotation (DCTA) Workflow**: Is a set of scripts to download H5AD files, execute the *HRApop CTann Tool Containers* (see **Methods** section), and output* CT* *populations* as well as donor metadata as input for the RUI2CTpop Workflow.
- **Donor**: Is an individual person, living or deceased, who contributed tissue. *Donors* have demographic metadata, such as age, BMI, race/ethnicity, and sex. All *donors* in HRApop are healthy, human adults.
- **Enrichment**: Refers to the process of augmenting existing data with additional information, metadata, or contextuallinks in order to increase its interpretability, usability, interoperability, and long-term value.
- **Experimental dataset**: Describes cell-by-gene matrices (via *CTann tools*) or cell-by-protein matrices^137^ derived from a *RUI-Registered* *tissue block* from a *donor*.
- **HRApop Atlas Data (also called HRApop Data Used in Atlas Construction)**: Is a collection of high-quality *experimental datasets* that fulfill *Criteria C1-4* that is used in atlas construction. Also part of the *HRApop Atlas* are the *CT populations of* 3D extraction sites of these datasets, plus the ASs with which these extraction sites collide, starting with a programmatically compiled collection of dataset IDs, extraction sites, and *donor* metadata that forms the input of the *DCTA Workflow*. Each HRApop run provides a series of reports (see GitHub^115^).
- **HRApop CTann Tool Containers**: Is a collection of CTann tools containerized with Docker to annotate H5AD files during the DCTA Workflow.
- **Intersection percentage or volume**: Is a measure that describes the shared 3D space between two 3D objects (e.g., a *tissue block* and an *AS*) expressed as a percentage of the total volume or as an absolute value in cubic millimeters).
- **Mean biomarker expressions**: Are computed for the top-10 genes per CT per dataset, then stored in the *DESpop*.
- **RUI to Compute CTpop (RUI2CTpop) Workflow**: Is a collection of scripts to compute the *HRApop Atlas Data* from the output of the *DCTA Workflow* (see Fig. 1**and Methods**).

### Computation

**HRApop** was computed with two automated workflows:

1. The **Download and Cell Type Annotation (DCTA) Workflow** (see GitHub^83^) used scalable, open-source software to programmatically download and annotate H5AD files from four portals: HuBMAP, SenNet, GTEx, and CELLxGENE. Cells in these H5AD files were then annotated with containerized CTann tools kept in the **HRApop CTann Tool Containers** (see GitHub^84^); next, the DCTA Workflow crosswalked the resulting CTann labels to CL^76^ or Provisional Cell Ontology (PCL)^85^, see the **Crosswalks** section for details, then compiled donor metadata from each dataset and finally made the result available as a set of CSV (for metadata) and JSON files (for CT populations). These were then used as input for the subsequent workflow.
2. The **RUI to Compute CTpop (RUI2CTpop) Workflow** (see GitHub^86^) computed CT populations for ASs, datasets, and extraction sites. It first identified all datasets that could be used to compute HRApop based on four Criteria (C1-4):
  **C1:** The dataset had a 3D extraction site by registration with the RUI (see **Methods**), i.e., the spatial position, rotation, and size of the tissue block (from which the dataset had been derived) within the HRA reference system is known and AS tags exist.
  **C2:** The dataset had a CT population. For sc-transcriptomics data, this meant having an associated H5AD file with a cell-by-gene matrix that could be annotated via Azimuth^50^, CellTypist^51,52^, and/or popV^53^; for sc-proteomics data, CT populations were obtained via manual segmentation and annotation workflows.
  **C3:** The dataset was of high quality, i.e., it had been downloaded from a data portal with built-in quality assurance/quality control or was associated with a peer-reviewed publication.
  **C4:** The dataset had donor metadata, was from a healthy tissue sample, and had an age value greater than 18. That is, the data was from a healthy adult human.

**C1** and **C2** were required to fill 3D AS with cells from one or multiple CTann tools. **C3** ensured that atlas data came from reliable sources. **C4** ensured a healthy, adult reference for HRA User Story #3 (compare reference tissue with aging/diseased tissue)^36^.

For datasets that met **C1-4**, RUI2CTpop output the **HRApop Atlas Data** (also called “**HRApop Data Used in Atlas Construction**”). Datasets that did not meet C1-4 were disregarded.

Figure 1 shows the computation and usage of HRApop, from the data download on the left to the publication and usage of *HRApop Atlas Data* on the right.

First, in the **DCTA Workflow**, datasets represented as H5AD files were programmatically downloaded from the four data portals (Fig. 1a) alongside donor metadata (Fig. 1b). Before download, non-human and diseased data were filtered out. Then, each dataset was annotated using all applicable CTann tools (Fig. 1c), resulting in CT populations if the dataset originated from a supported organ (Fig. 1f). Only if a dataset met all Criteria C1-4, then it was used for HRApop Atlas construction.

Next, in the **RUI2CTpop Workflow**, datasets were matched against all existing RUI extraction sites by their ID (Fig. 1d**)**; the extraction sites held metadata on organ sex and laterality (left or right) and could come from either an API (such as for HuBMAP or SenNet, see **HRA registrations and extraction sites via APIs** section) or the static HRA Registrations^87^ (see Table S1), a manually curated collection of extraction sites.

For each HRApop Atlas Dataset, the 3D ASs that its extraction site collided with were determined via mesh-based *collision detection* (Fig. 1e). The example shown is the renal pyramid (purl.obolibrary.org/obo/UBERON_0004200) of the left, male kidney (lod.humanatlas.io/ref-organ/kidney-male-left/latest) with two hypothetical tissue blocks colliding with it at 75% and 50% of the total volume of the extraction site. The number and percentage of CTs that should be in the colliding AS based on the intersection percentage of the extraction site were computed (see Fig. 1g). Typically, many tissue blocks from different donor demographics (e.g., age, ethnicity) existed per male/female-specific ASs. As research teams carefully sample from the very same RUI extraction site^36^, *intersection percentage* computation was performed for every extraction site that intersected with the AS. The result is an ASpop containing the unique and shared CTs contributed by each colliding extraction site (see Fig. 1h).

Then, the ASpop (and the DESpop used to generate the ASpop) were published as two separate Resource Description Framework (www.w3.org/RDF) graphs (see Fig. 1i) via the HRA KG^21^ at lod.humanatlas.io/graph/hra-pop/latest in support of Linked Open Data principles. Other data products are described under **Data Records**.

Finally, the outputs from the RUI2CTpop Workflow were made available for usage in various HRA UIs and the HRA API (apps.humanatlas.io/api, Fig. 1j). HRA KG queries can be run to support diverse applications inside and outside the HRA data ecosystem. Examples for applications using HRApop data are the HRA Functional Tissue Unit Explorer (apps.humanatlas.io/ftu-explorer), the HRA Organ Gallery in virtual reality^88,89^, and the HRApop Visualizer; further, ad-hoc queries, such as one that provides an overview of all AS-CT combinations, including sex, tool, cell count, and cell percentage, available at apps.humanatlas.io/api/grlc/hra-pop.html#get-/cell_types_in_anatomical_structurescts_per_as.

An exemplary ASpop from the kidney and an exemplary snippet of the DESpop are available on the companion website at cns-iu.github.io/hra-cell-type-populations-supporting-information#exemplary-cell-type-populations. A complete listing of all data and code for computation and usage of HRApop is provided in Table S1. An overview of all HRA applications that use **HRApop Atlas Data** is provided in Table S2.

### HRA registrations and extraction sites via APIs

For all extraction sites, *mesh-based collision detection* was used to compute the intersection percentage with 3D ASs, which enabled the computation and aggregation of CT populations for these 3D ASs. Where possible, for all datasets, extraction sites, and ASs, HRApop provided CT populations from every tool that could annotate the dataset. All extraction sites used in the HRApop Atlas Data are shown in the HRApop-focused Exploration User Interface^25^ (EUI) at cns-iu.github.io/hra-cell-type-populations-supporting-information/eui.html. By working closely with authors of published, high-quality datasets, as well as tissue providers in HuBMAP and SenNet, RUI was used to generate extraction sites for a growing number of organs and ASs.

Table 1 presents counts for datasets, extraction sites, ASs, and organs based on input data for RUI2CTpop Workflow and the HRApop Atlas.

**Table 1:** Number of datasets, extraction sites, ASs, and organs covered in HRApop v1.0.

	Datasets	RUI extraction sites	ASs covered	Organs covered
**Input data for RUI2CTpop Workflow**	16,293	1,132	164	49
**HRApop Atlas**	662	230	73	17

### Experimental data

**Fig. 2:**
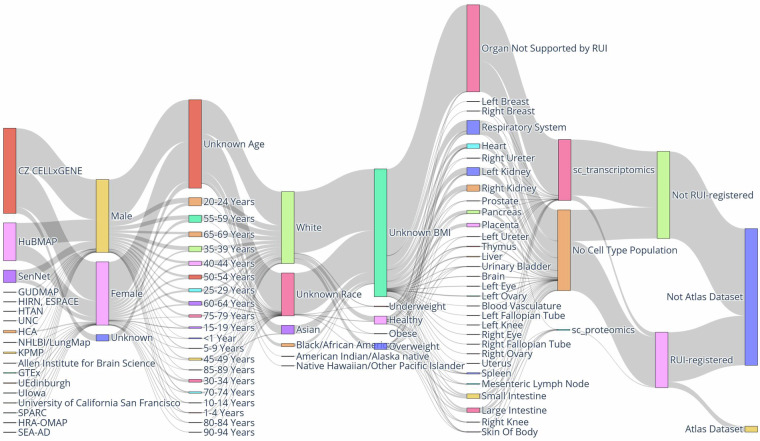
Sankey diagram of all input data for the RUI2CTpop Workflow. Note the Atlas Dataset node in the bottom right corner, which represents the 662 HRApop Atlas Datasets in HRApop v1.0.

The Sankey diagram in Fig. 2 provides a high-level overview of the input data for the RUI2CTpop Workflow along several axes. It can be explored interactively at cns-iu.github.io/hra-cell-type-populations-supporting-information/sankey_universe_plotly.html. A version showing only HRApop Atlas Data is shown in Fig. S2, with an interactive version available at cns-iu.github.io/hra-cell-type-populations-supporting-information/sankey_atlas_plotly.html.

The Sankey diagram has nine vertical axes that represent:

#### Portal/source

Identifies the effort where the data originated. H5AD files were downloaded from HuBMAP, SenNet, GTEx, and CELLxGENE. The majority of datasets came from CELLxGENE. All other portals/sources were derived from extraction sites.

#### Donor sex, age, race, BMI

Describes clinical metadata for the human specimens from which the data was retrieved. Where available, donor age was provided as an integer by HuBMAP and SenNet but as string by CELLXGENE (e.g., “61-year-old human stage”); as a result, string literal age values for CELLxGENE data were parsed as a number where possible. To enable visualization in a Sankey diagram, all age values were aggregated into bins of five years. For race, the same categories were used as on the HuBMAP Data Portal (portal.hubmapconsortium.org). BMI values were mapped to categories by brackets defined by the Centers for Disease Control on www.cdc.gov/bmi/adult-calculator/bmi-categories.html.

#### Organ

Indicates the organ of origin. “Organ Not Supported by RUI” means that there was no matching 3D reference object in the HRA (e.g., for blood).

#### CT population

Means that a dataset had either (1) one or multiple CT populations from one or multiple CTann tools (“sc_transcriptomics”), (2) no CT population because no CTann tool existed for the dataset (“No Cell Type Population”), or (3) a CT population via sc-proteomics as a generalization of the HRApop workflow (“sc_proteomics”).

#### Extraction site

Indicates whether a dataset had an extraction site via RUI registration or not.

#### HRApop Atlas Data

Indicates whether the dataset was part of the HRApop Atlas or not. This was only true for 662 datasets.

### Computational resource requirements

For both the DCTA and RUI2CTpop workflows, computational hardware with 10 or more cores, 256GB RAM, and at least 4TB of disk space is recommended, with a Linux-based environment like Ubuntu as the operating system of choice. For the DCTA workflow, annotations per dataset can be distributed among similarly configured servers. For HRApop v1.0, five Linux servers running 10 dataset annotation runs per server in parallel for a maximum of 50 runs executing in parallel were used.

The DCTA Workflow for HRApop v1.0 started on Thu, May 15, 2025, ran for about 10 days, and finished on Sunday, May 25, 2025. It averaged 87.63 dataset annotation runs per hour. Annotations took about 8.59 days to finish. After a 22-day QA phase, the RUI2CTpop Workflow started on June 16 at 5:55:27 PM EDT and finished about four hours later at 10:07:11 PM EDT the same day. A full log is linked in Table S1.

### Data used

A table on GitHub^90^ lists dataset IDs for all 16,293 datasets originally downloaded on May 15, 2025, for HRApop v1.0. Below, the number of public datasets available on four portals are provided for January 2026.

### Data portals

#### HuBMAP

As of January 2026, the HuBMAP Data Portal (portal.hubmapconsortium.org) listed 5,250 public datasets from 28 organs. These datasets were ingested by tissue providers through an Ingest UI (ingest.hubmapconsortium.org) where they could enter donors, organs, samples/tissue blocks, tissue sections, etc. Relationships between these entities are organized in a provenance hierarchy where a donor and organ are needed so that tissue samples can be organized based on diverse tissue sample types. APIs enable users to access entities programmatically (docs.hubmapconsortium.org/apis.html). Both published and unpublished datasets exist on the HuBMAP and SenNet portals (see below). Published datasets have been sent through a series of quality assurance/quality control processes. Unpublished datasets are only accessible with authentication. Only published datasets were used for HRApop construction. Concatenations of HuBMAP data organized by assay and organ exist at data-products.hubmapconsortium.org/data_products.

#### SenNet

The SenNet Data Portal (data.sennetconsortium.org/search) uses a similar infrastructure as the HuBMAP Data Portal. It features human and murine datasets. As of January 2026, 1,298 human datasets from 234 donors were publicly available. Like for HuBMAP, APIs provide programmatic access to datasets, donors, organs, etc., see docs.sennetconsortium.org/apis.

#### GTEx

The GTEx Portal (www.gtexportal.org/home/downloads/adult-gtex) hosts the adult GTEx data and resources and provides open access to, e.g., expression quantitative trait loci, and protected access, e.g., to limited donor phenotypes as well as de-identified donor data for sequencing. For GTEx sc-data, one H5AD file with data for all 8 organs plus donor metadata was downloaded (storage.googleapis.com/adult-gtex/single-cell/v9/snrna-seq-data/GTEx_8_tissues_snRNAseq_atlas_071421.public_obs.h5ad).

#### CELLxGENE

The CELLxGENE Portal (cellxgene.cziscience.com/collections) provides access to both primary and secondary datasets. Primary datasets contain the raw or minimally processed data while secondary datasets are curated and normalized. The DCTA Workflow retrieved the secondary datasets when possible unless they contained fewer data points than the primary datasets. As of January 2026, there were 212 collections from healthy donors older than 15 years.

### Cell counts

For sc-transcriptomics data, cell counts represent the unprocessed number of RNA transcripts detected for each gene in each cell. To ensure data integrity and consistency, raw cell counts were used wherever available during CTann. For HuBMAP and SenNet datasets, these were obtained from the *counts* layer of the H5AD file, which contains integer counts per gene and cell, while for GTEx and CELLxGENE datasets, raw counts were obtained from the *raw.X* attribute of the H5AD file, which stores original and unnormalized counts for each cell and gene. For sc-proteomics data, cell counts were obtained by counting the number of rows in the cell tables provided by the data providers.

### Crosswalks

To enable comparisons between CTs assigned by different CTann tools (and sc-proteomics data, which used human-assigned CTs), CT labels needed to be crosswalked to CTs in the anatomical structures, cell types, plus biomarkers (ASCT + B) tables^37^ using CL or PCL terms. Crosswalks for each CTann tool were curated manually by experts and are published at lod.humanatlas.io/ctann; the underlying HRA Digital Object type is described in a related publication^21^. Since not all CTs had an exact match to a CL term, the Simple Knowledge Organization System (www.w3.org/2004/02/skos)^91,92^ was used to indicate if the mapping was done to a term with an exact match (*skos:exactMatch*) or if they had to be mapped to a more general class (*skos:narrowMatch*). The crosswalks published in HRA v2.3 linked 1,615 annotation labels and 1,909 annotation IDs from Azimuth, CellTypist, popV, and sc-proteomics datasets (author-assigned) to 885 CL labels and 495 CL IDs for 36 organs. 1,923 mappings were exact matches and 683 were narrow matches.

The DCTA Workflow applied crosswalks after CT annotations were done; CL labels and IDs were used when computing CT populations for ASs, datasets, and extraction sites.

### Existing code

#### CTann tools

The three CTann tools in HRApop v1.0 (Azimuth^50^, CellTypist^51,52^, popV^53^) were containerized with Docker in the *HRApop CTann Tool Containers* on GitHub^84^. The Dockerfile for each container listed operating system requirements, basic setup, and dependencies so the package could be run by the DCTA Workflow in the cloud or on a local machine with consistent outputs. These containers defined a dataset handler interface (see code on GitHub^93^) that specified requirements for every new piece of code to download H5AD files from a data portal. Apptainer (apptainer.org) was used when running HRApop code on HPC clusters. An exemplary context file is provided in this example^94^ for Azimuth. Similarly, extracting CT populations, computing gene expressions, and crosswalking were also packaged as Docker files. Both Docker and Apptainer support running Common Workflow Language (CWL, www.commonwl.org) workflows on a Linux cluster for HRApop v1.0. The entire container setup for Azimuth is available on GitHub^95^. Table S3 lists the name, version number, code base, models used, and requirements for each tool.

#### Mean biomarker expressions for sc-transcriptomics data

*Mean biomarker expressions* were captured in the DESpop and provided for each CT per dataset. To generate these values, *scanpy*^96^, *numpy*^97^, and *anndata*^98^ were used. Specifically, *scanpy*’s *rank_gene_groups()* method (scanpy.readthedocs.io/en/stable/generated/scanpy.tl.rank_genes_groups.html) was applied to perform differential expression analysis between CTs. As part of this analysis, this method calculated the mean expression of each gene within a target CT, as well as its expression in the rest of the dataset. These values were used to identify and rank marker genes, and the corresponding mean expressions were recorded for the top-n genes, where n was defined by the user. However, it is important to note that this method does not compute mean expression values for all genes across all CTs—only for the most differentially expressed ones. To ensure consistency in gene naming across datasets, gene identifiers were normalized using a lookup table, which maps Ensembl IDs from Release 111^99^ (www.ensembl.org/index.html) to HGNC-approved symbols from version v2023-09-18^100^ (www.genenames.org). This normalization helps maintain interoperability and accuracy when comparing gene expression data across datasets and tools.

#### Tissue registration

Registering tissue datasets inside the 73 3D reference objects in the HRA v2.3 was made possible via the RUI^25^, which generates cuboid, 3D extraction sites. The RUI is available as a standalone tool at apps.humanatlas.io/rui but also embedded into the ingest pipelines of the HuBMAP and SenNet data portals. The registration process consisted of three main phases: assignment, *enrichment*, and validation. Initially, the registration coordinator contacted subject matter experts with knowledge of the spatial information associated with tissue samples. Experts could then use the RUI to submit spatial information for tissue blocks themselves or collaborate with the coordinator, who facilitated the submission process with their input. The RUI recorded spatial information by creating extraction sites inside 3D reference objects. Additionally, it used mesh-based collision detection to annotate the extraction with AS tags (see **Mesh-based collision detection** section). By the end of this phase, all tissue samples had an assigned extraction site. These workflows are further detailed in standard operating procedures^27–29^.

Once extraction sites were assigned, enrichment began. The registration coordinator used a location processor tool to enhance spatial information with de-identified donor metadata (e.g., sex, age, BMI) and publication metadata (e.g., DOI, authors, publication year). This combined dataset formed a registration set^36^, which was assigned a unique ID for future reference. The code for the processor is accessible via GitHub^101^, as are existing stand-alone HRA Registrations^87^.

Finally, in the validation phase, the expert was asked to review the registration set for accuracy and completeness, with a request for revisions if necessary. This process used a customized instance of the EUI^25^, which the expert used to evaluate sample locations, metadata accuracy, and AS tags from mesh-based collision detection. Once validated, the registration set was finalized and added to the general EUI (apps.humanatlas.io/eui). This concluded the spatial registration process. An overview of all EUIs for registration sets is available on GitHub^102^.

#### Mesh-based collision detection

To enable more precise collision detection between tissue blocks and ASs, a library for mesh-based collision detection was created and named HRA Mesh Collision API^103^. Given an extraction site, this HTTP service returns a list of mesh collisions with ASs and metadata. The 3D geometry-based tissue block annotation code includes: (1) a C++ library for the HTTP service for collision detection and *intersection* *volume* computation between extraction sites and ASs, (2) a C++ library for checking manifoldness and closedness of meshes as well as hole-filling for unclosed meshes, and (3) a Python library for converting binary Graphics Library Transmission Binary Format (GLTF/GLB, www.khronos.org/gltf) files to Object File Format (OFF) files, used as the underlying 3D model format for collision detection. The code repository, URL to deployed API, and exemplary API response are available in Table S1.

#### Weighted cosine similarity

A collection of functions to compute and use weighted cosine similarities in the RUI2CTpop Workflow is available on GitHub^104^. The script uses math.js (mathjs.org) for access to implementation for the dot product and norm between two vectors (mathjs.org/docs/reference/functions/dot.html and mathjs.org/docs/reference/functions/norm.html).

### New Code

#### DCTA workflow

Downloading and annotating datasets was handled by the **DCTA Workflow** (see GitHub^83^). First, it ensured that the data from multiple portals was from healthy, human donors; ran applicable CTann tools via the **HRApop CTann Tool Containers**; analyzed gene expressions to identify top genes with *scanpy*; crosswalked CT labels from CTann tools to ASCT + B tables^37^ using crosswalks; assembled donor metadata; and output summarized results for downstream use. It was runnable as a CWL workflow. The CWL runner was written in Python. The DCTA Workflow then produced CT populations and metadata for all annotated sc-transcriptomics and sc-proteomics datasets as output (see Table S1). They were then copied to the input GitHub repository^105^ for the RUI2CTpop Workflow (see below) for further processing.

#### Download

To download H5AD files, the DCTA Workflow constructed a series of jobs to execute. An organ mapping provided crosswalks between organ code names on the data portals forHuBMAP and SenNet (see this GitHub commit^106^) as well as GTEx (see this GitHub commit^107^) to Uberon IDs and labels. For CELLxGENE, no mapping was needed as the metadata contained Uberon IDs already. To retrieve donor metadata across portals, each portal was queried through their APIs, then relevant information was extracted and saved in a harmonized format, see donor field at this GitHub commit^108^. Implementations to extract donor metadata from the different portals are also available, e.g., for SenNet (see this GitHub commit^109^) and CELLxGENE (see GitHub^110^).

The DCTA Workflow extracted metadata needed for constructing ds-graphs^21^ (age, sex, BMI, assay type) from the individual portal APIs and saved it as JSON files. The H5AD files were downloaded locally into a raw data folder in the DCTA Workflow repository, or onto a file system on a HPC system.

#### Splitting and re-assembling H5AD files for GTEx and CELLxGENE data

In the data model of HuBMAP and SenNet, donors, organs, tissue blocks, tissue sections, and datasets are modeled as individual entities, where each dataset belongs to a single donor. This means that an H5AD file from HuBMAP or SenNet contains data for exactly one donor. On CELLxGENE, on the other hand, H5AD files contain multiple donors; to make the two data models work together, H5AD files from CELLxGENE were split into new H5AD files by donor and organ in the DCTA Workflow. The respective script^111^ was written in Python, because it needed *pandas*, a foundational library for data manipulation and analysis (pandas.pydata.org), and *anndata* for handling annotated data matrices (anndata.readthedocs.io/en/stable). This was done to combine donor-organ combinations across assets into new H5AD files. Extracted donor metadata fields were shown in the harmonized donor metadata format described above. These new H5AD files are made available alongside all other H5AD files used for computing HRApop v1.0 on Globus^112^.

#### Datasets with too few cells

Datasets with fewer than 100 cells were filtered out by the DCTA Workflow. While their H5AD files were downloaded, no CTann tool was run over them and no CT population was computed.

### RUI2CTpop Workflow

The **RUI2CTpop Workflow** (see GitHub^86^) performed spatial annotation and CT population computation with input files provided by the DCTA Workflow. It sourced extraction sites via the HuBMAP and SenNet APIs through HRA API queries at apps.humanatlas.io/api#get-/ds-graph/hubmap, apps.humanatlas.io/api#get-/ds-graph/sennet, and apps.humanatlas.io/api#get-/ds-graph/gtex, with the underlying queries at github.com/x-atlas-consortia/hra-api/blob/main/src/library/ds-graph/operations/hubmap.js, github.com/x-atlas-consortia/hra-api/blob/main/src/library/ds-graph/operations/sennet.js, and github.com/x-atlas-consortia/hra-api/blob/main/src/library/ds-graph/operations/gtex.js. A listing of all sources for extraction sites is available at github.com/x-atlas-consortia/hra-pop/blob/main/input-data/v1.0/config.sh.

For the RUI2CTpop Workflow to function, the DCTA Workflow provided CT populations and dataset metadata, then those files were copied over to the input folder for a new RUI2CTpop run (see GitHub^70^). Scripts running over these input files during RUI2CTpop are on GitHub^113^. Output data from **RUI2CTpop** is provided on GitHub^114^.

The RUI2CTpop Workflow processed Criteria C1-4 (including a check for donor age to ensure only data from adult humans is used) and gathered extraction sites, CT populations, donor metadata, and related publications where applicable. If a dataset had no metadata for age or sex, it was not used for atlas construction. GTEx provided only age ranges, not values, but the data came from adult donors. The RUI2CTpop Workflow then used mesh-based collision detection to build the ASpop and the DESpop (see links in Table S1) and code to compute *corridors* (see **Corridors** section). Exemplary CT populations for a dataset, an extraction site, and an AS are shown at cns-iu.github.io/hra-cell-type-populations-supporting-information/#exemplary-cell-type-populations. Note that in all three cases, the CTann tool(s) are indicated by the *annotation_method* field. The RUI2CTpop Workflow also contained scripts and SPARQL queries to construct data products for HRApop in the form of CSV reports^115^ to analyze, visualize, validate, and use HRApop data (see link in Table S1).

### Corridors

For each extraction site with a CT population, a 3D volume of likely origin within the organ was computed, given the biomolecular make-up of the tissue block as represented by its CT population. The result was a complex corridor, i.e., a combined representation for all possible locations, compiled via alpha wrapping with an offset^116^. Corridors represented the complete set of spatial positions where extraction sites could plausibly be located while maintaining their observed intersection ratios with neighboring ASs. Each extraction site was uniquely associated with one such corridor. Corridors were GLB files (see **Data Records**). The spatial origin could be an entire AS if it had the same or a similar CT population (measured using weighted cosine similarity) or the extraction site of a tissue block with the most similar CT population (and its corresponding corridor with the same percentages of multiple ASs).

To generate complex 3D corridors given an extraction site with the RUI, a C+ + library with an HTTP service for the 3D Corridor Generation API^117^ was created (apps.humanatlas.io/api/#post-/v1/corridor, see also Table S1). Corridors were computed by sending an extraction site to this API. From there, three cases are possible: (1) If the extraction site collided with **only one AS**, the entire AS was returned as a corridor; (2) if the extraction site collided with **two ASs**, a filter-search algorithm was used to compute all the possible locations before applying alpha wrapping with an offset to generate a complex corridor. The filter-refine paradigm^118^ is widely used in computationally intensive tasks such as the one presented here, where infeasible solutions are filtered out from a list of candidates. Next, more viable candidates are examined with respect to their exact geometry to generate exact answers in a refinement step. Inspired by the filter-refine paradigm, a filter-search algorithm was made to derive complex corridors. Finally, (3) if a tissue block collided with **three or more ASs**, it was fixed in place, in which case it corresponded exactly to the extraction site.

Corridors were made available as a ZIP file on Zenodo^119^; they were named after the extraction site on which were based, e.g., corridors/1cbd9283-2d58-4a2d-88fe-effb18c3f14f.glb, which belongs to the extraction site with ID 1cbd9283-2d58-4a2d-88fe-effb18c3f14f from the head of the female pancreas. It can be inspected in 3D on the companion website at cns-iu.github.io/hra-cell-type-populations-supporting-information#exemplary-corridor. HRApop v1.0 made 1,189 corridors available, including the 230 corridors for the 230 extraction sites in DESpop, plus 959 corridors for extraction sites not in the HRApop Atlas. Their total size is 202 MB (99.8 MB when compressed). Code, endpoint, and documentation for the 3D Corridor Generation API are available in Table S1.

### Tradeoff between step size in search stage and precision of corridor

Since a sliding window approach was used to search feasible locations if two or more AS collided with an extraction site, configuring the step size was essential. It determined how big a move was made in the search stage (see Table 2). If a large step size was set, locations with the exact intersection volume with the given extraction site could be skipped. Conversely, if a small step size was set, the computation cost could become overwhelming. Further, the intersection volume from the mesh-based collision detection was returned as a float; thus, if only the exact value was matched, there could be very few or no feasible locations. In order to compute corridors with both high precision and reasonable computational overhead, the tolerance for feasible locations had to be adjusted to, e.g., 0.1, which means the difference between the intersection volume of the feasible locations and the true intersection volume could not exceed 10% of the true intersection volume.

**Table 2:** Filter and search stage for pre-computing corridors.

1	**Filter stage**	Each collided mesh was approximated by its minimum bounding boxes. Then, the area *Ω*, where a fixed-size, axis-aligned tissue block could be put, was computed so that it could intersect with all the minimum bounding boxes of meshes.
2	**Search stage**	A brute force search algorithm was applied by specifying the step size. A sliding window approach was used to move the fixed-size tissue block within the search area *Ω* that was computed in the filter stage.

## Data Records

The processed data and archived code are available on Zenodo, while raw data is on Globus (www.globus.org) and actively developed code is on GitHub.

### HRApop data products on Zenodo

Six major HRApop data products are available for download on Zenodo^119^:

1. HRApop Atlas Data v1.0 (covers 73 ASs in 17 organs via 662 HRApop Atlas datasets: 558 sc-transcriptomics, 104 sc-proteomics)
  - ASpop (JSON-LD, see json-ld.org).
  - DESpop (JSON-LD).
2. Input for the RUI2CTpop Workflow.
  - *Cell instances*, i.e., occurrences of unique cells, with top 10k genes for all datasets downloaded for the DCTA Workflow (compressed CSV).
  - Corridors for all extraction sites used as input for the DCTA Workflow (ZIP folder with GLB files). Contains 1,189 corridors, including 230 corridors in DESpop.
3. QC
  - Cell instances with confidence scores for each CTann tool assignments (compressed CSV), used for Fig. 4 in the **Technical Validation**.
  - QC metrics for all sc-transcriptomics datasets that are annotated via the DCTA Workflow are provided as a ZIP file with one folder per dataset, used for Table S6 as well as Fig. 5 in the **Technical Validation**.

### Direct download links for CSV files

Table 3 below provides links to download ASpop and DESpop as CSV files.

**Table 3:** Direct links to CSV files for ASpop and DESpop.

Name	URL	Type
CT populations for ASs	apps.humanatlas.io/api/grlc/hra-pop/cell_types_in_anatomical_structurescts_per_as.csv	ASpop
CT populations for extraction sites	apps.humanatlas.io/api/grlc/hra-pop/cell-types-per-extraction-site.csv	DESpop
CT populations for datasets	apps.humanatlas.io/api/grlc/hra-pop/cell-types-per-dataset.csv	DESpop

### Raw data on Globus

All H5AD files used for computing ASpop and DESpop from sc-transcriptomics and sc-proteomics datasets are provided for download on Globus^112^. The HuBMAP Command Line Transfer utility (docs.hubmapconsortium.org/clt) enables the user to download the content of the */extras* folder in the Globus directory associated with this publication, see “Bulk Data Transfer” at portal.hubmapconsortium.org/browse/publication/f53d60b5994333777a446dd7ad3b0304 (HuBMAP ID: HBM536.HGTK.934). To then check for batch effects in the raw H5AD files, packages like kBET^120^, LIGER^121^, or Seurat^122,123^ can be used.

### Archived code

The release code for HRApop v1.0 was archived on Zenodo for the DCTA Workflow^124^, the HRApop CTann Tool Containers^125^, and the RUI2CTpop Workflow^126^.

### Miscellaneous

A full listing of repositories used to construct and use HRApop v1.0 is provided in Table S1. Examples for usage of this HRApop Atlas Data are available on the companion website at cns-iu.github.io/hra-cell-type-populations-supporting-information#usage-examples. The **Usage Notes** section details how to access HRApop data via Jupyter Notebooks.

## Data Overview

### Counts for HRApop v1.0

On June 16, 2025, the RUI2CTpop Workflow was run to compute HRApop v1.0. It downloaded 16,293 datasets with 57,911,931 cells from the four sc-portals, which were then sent through a filtering process.

558 of the 662 datasets in the HRApop Atlas Data were sc-transcriptomics datasets that were annotated using Azimuth^50^, CellTypist^51,52^, and/or popV^53^, covering 11,042,750 unique cells in a total 3D volume of ~12.05 liters (dm³) with partially intersecting extraction sites in 73 unique ASs in 17 organs (112 ASs and 31 organs if male and female are counted separately). The datasets came from 230 3D extraction sites that covered 54 3D ASs across 17 organs. While the HRApop Atlas focuses on these 558 sc-transcriptomics datasets in 17 organs, the method is generalizable to sc-proteomics (see **Generalization to spatial data** section) and to all organs.

Table 4 provides counts of datasets and cells in the HRApop Atlas Data, split by sex, consortium, number of datasets, number of cells, and modality. HuBMAP, SenNet, and GTEx have their own portals. Human Cell Atlas and NHLBI/LungMap^127,128^ datasets all come from the CELLxGENE Portal. Detailed counts for the HRApop Atlas from the RUI2CTpop Workflow are provided in Table S4.

**Table 4:** HRApop Atlas Datasets.

Sex	Consortium	#Datasets	#Cells	Modality
Female	GTEx	7	47,863	sc_transcriptomics
Male	GTEx	8	70,113	sc_transcriptomics
Female	Human Cell Atlas	63	364,993	sc_transcriptomics
Male	Human Cell Atlas	58	359,273	sc_transcriptomics
Female	HuBMAP	22	900,547	sc_proteomics
Female	HuBMAP	112	2,933,015	sc_transcriptomics
Male	HuBMAP	82	15,676,316	sc_proteomics
Male	HuBMAP	253	6,626,620	sc_transcriptomics
Female	NHLBI/LungMap	1	4,680	sc_transcriptomics
Male	NHLBI/LungMap	2	5,713	sc_transcriptomics
Female	SenNet	18	225,940	sc_transcriptomics
Male	SenNet	36	404,540	sc_transcriptomics
**TOTAL**		**662**	**27,619,613** sc-transcriptomics: **11,042,750** sc-proteomics: **16,576,863**	

A breakdown of datasets that meet Criteria C1-4 and are used to construct HRApop v1.0.

### Anatomical structure cell type populations

**Fig. 3:**
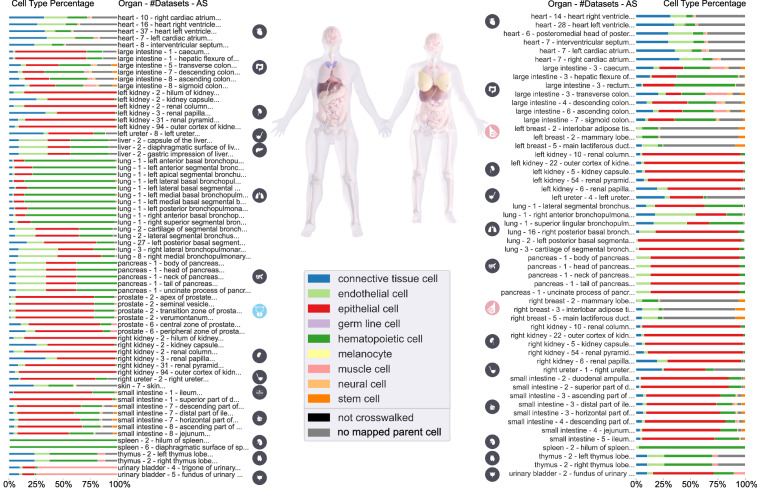
CT populations for unique ASs across male and female in HRApop v1.0. Stacked bar graphs of the percentage of CT identified in ASs, aggregated to higher level CTs from CL (see listing on GitHub^135^), are shown for male (left) and female (right; note that the 3D placenta was omitted from the rendering of the two bodies in the center). The male-only organ icon (prostate) is rendered in blue while icons for female-only organs is rendered in pink (left and right breast).

Figure 3 shows the 112 ASs of the male (left) and female (right) reference body for which spatially registered sc-transcriptomics data existed in HRApop v1.0. For each AS, the organ name, number of datasets, and AS name plus a bar graph with the percentage of major CTs, i.e., ASpop, are shown. Only sc-transcriptomics data is shown and Azimuth^50^, CellTypist^51,52^, and/or popV^53^ were used to annotate cells, with a preference for Azimuth annotation (if present) over CellTypist (if present) over popV^53^. Crosswalking to CL or PCL and aggregation to higher-level CTs is also described in detail in the **Crosswalks** section. For the left and right breast, two CTs that cannot be mapped to a high-level CT make up ~70% of the CTs across ASs. As a result, the stacked bar graphs for these are mostly grey: luminal epithelial cell of mammary gland (purl.obolibrary.org/obo/CL_0002326) with ~53% and fibroblast of breast (purl.obolibrary.org/obo/CL_4006000) with ~16%.

Data featured in Fig. 3 used CTann crosswalks to harmonize and compare datasets across portals. The resulting CT typology has 201 low-level CTs that occur in the experimental datasets in HRApop v1.0. CL defines 11 broad, high-level classes — a set curated by CL editors to cover most low-level CTs while minimizing overlap; it is available on GitHub^129^. The 201 CTs in HRApop v1.0 fall into only 9 of these classes, leaving two unpopulated (extraembryonic cell and bone cell). Additional mappings exist to 19 medium-level, more granular CTs—a subset of terms that, while still broad, provides a more detailed classification via CL IDs and labels; this mapping is available in Table S1. The mapping applied in Fig. 3 used the 9 CTs belonging to the upper slim of high-level CTs directly from CL (see GitHub^129^); note that CTs from PCL are included in this slim ontology. 26 CTs were classified under more than one category in CL; for these, one of the classes reflecting expected biological grouping was chosen (see Table S5).

If a CT was marked as “no mapped parent cell,” the CT term (already crosswalked to CL or PCL) was not a subclass of a top-level CT in the top-n CTs provided by CL; in HRApop v1.0, a total of 617,000 cells associated with 10 CTs were marked gray. If a CT was marked as “not crosswalked,” this means that the CT label assigned by the CTann tool was not associated with a CL or PCL ID.; in HRApop v1.0, 9 cells associated with 1 CT in the small and large intestine that were not covered in existing ontologies and are rendered in black. A full report of cells that were not crosswalked while constructing HRApop but that have been crosswalked semi-manually for Fig. 3 was labeled “unmapped-cell-ids” and is available on GitHub^130^.

## Technical Validation

Four validations are presented in this section: for sc-transcriptomics data, we show (1) bins of confidence scores for each cell instance by tool, (2) ribosomal and mitochondrial gene percentages and counts aggregated by organ, and (3) the number of datasets per AS by organ and sex with available CTann tool(s); for sc-transcriptomics and sc-proteomics together, we show (4) heatmaps with different CT prevalence between AS in the same organ.

### Sc-transcriptomics data

#### Confidence scores per cell per tool

**Fig. 4:**
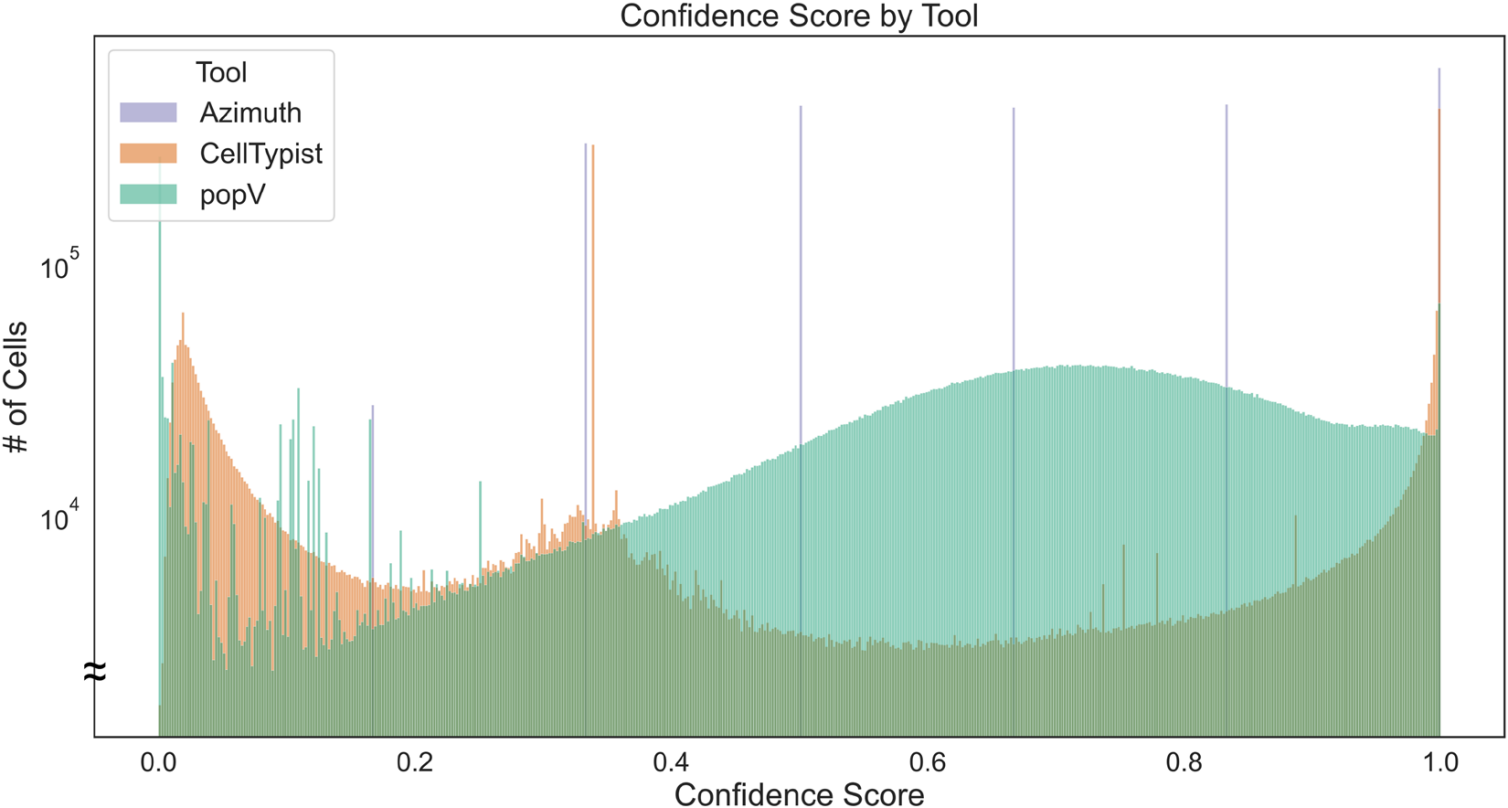
Histogram of confidence scores by number of cells with that score, using a bin width of 0.002, colored by CTann tool. Note that the y-axis is truncated for a more detailed view.

When running the DCTA Workflow, Azimuth, CellTypist, and popV computed confidence scores for each cell instance annotation. Figure 4 shows a histogram where the x-axis presents confidence scores (with a bin width of 0.002), the y-axis presents the number of cells per bin, and color encodes the tool assigning the confidence scores. The mean confidence scores are: Azimuth (mean = 0.62, median = 0.68, SD = 0.26), CellTypist (mean = 0.46, median = 0.34, SD = 0.37), popV (mean = 0.71, median = 0.67, SD = 0.24). The histogram shows that Azimuth and CellTypist have different values for their measures of central tendency while both generating continuous confidence scores, whereas popV generated spikes due to its averaged voting mechanism. For this paper, each tool has their own strengths and weaknesses, and as related work on benchmarking different CTann tools has shown^80^, when there is a discrepancy, there is no consensus on the best tool. This Data Descriptor provides results of a scalable, reproducible workflow that runs these three CTann tools at scale while providing the user with data products documenting the results so that they can apply their expertise to assess the CTann assignments. The code to reproduce the histogram in Fig. 4 is provided on GitHub^131^. The compressed CSV file is available on Zenodo^119^ (filename: “sc-transcriptomics-cell-instances.csv.gz”).

#### Ribosomal and mitochondrial gene percentages per organ

**Fig. 5:**
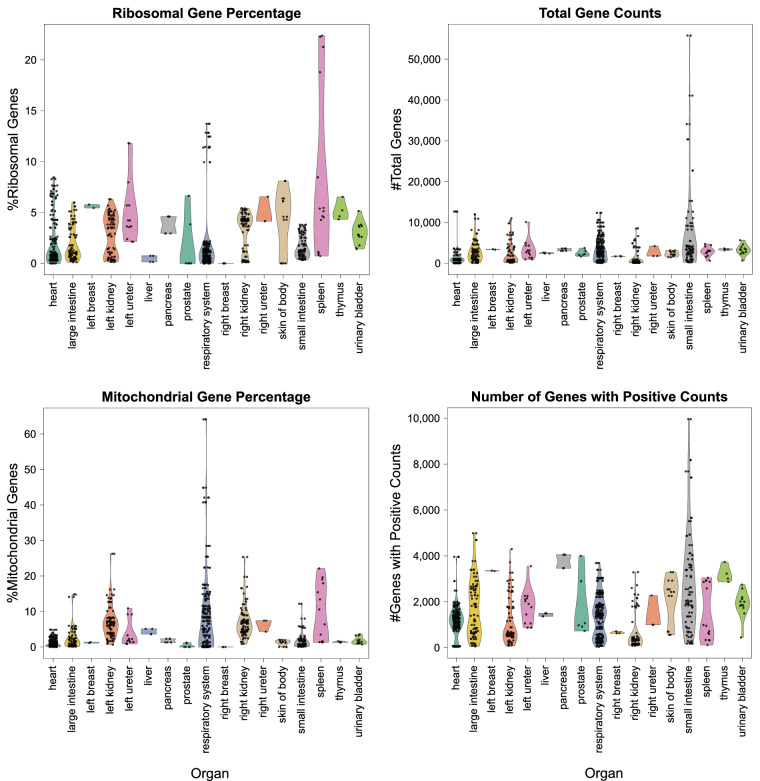
Violin plots with jittered dots for the ribosomal and mitochondrial gene percentage, plus genes with positive counts and total gene counts per organ.

Using *scanpy*’*s* built-in *calculate_qc_metrics()* function (scanpy.readthedocs.io/en/stable/generated/scanpy.pp.calculate_qc_metrics.html), mean and median ribosomal and mitochondrial gene percentages for all 558 sc-transcriptomics datasets (H5AD files) were computed and then aggregated to the mean and median at the organ level plus standard deviation, see Table S6. Meanwhile, Fig. 5 shows violin plots with jittered dots for the ribosomal and mitochondrial gene percentage, plus genes with positive counts and total gene counts per organ.

For QC purposes, we followed practical, commonly used QC ranges for ribosomal and mitochondrial gene percentages in single-nucleus and single-cell data. The thresholds for **ribosomal** gene percentages are less standardized than mitochondrial percentages, but there are practical norms used in QC (**10–40%** for single-nucleus, **5–30%** for single-cell). The thresholds for **mitochondrial** gene percentages are different (**1–3%** for single-nucleus, with **3–5%** being acceptable, and <**5–10%** for single-cell, with **10–15%** being acceptable). Note that these ranges differ heavily by CT and tissue. While the available metadata from HuBMAP and SenNet made it impossible to reliably capture whether a dataset was single-nucleus or single-cell, Fig. 5 and Table S6 show that mean and median percentage values are broadly within major single-nucleus and single-cell thresholds. This is to be expected, given that only datasets from portals with built-in quality assurance/quality control were used or datasets with an associated peer-reviewed publication. Finally, the mean number of genes with positive counts and mean total number of genes per organ are provided in Table S7.

A ZIP file with the QC results containing one directory per dataset is available on Zenodo^119^ (filename: “hra-pop-v1.0-qc.zip”). The code to reproduce Table S6, Fig. 5, and Table S7 is provided on GitHub^132^.

### Sc-transcriptomics and sc-proteomics data

#### Heatmaps

While some CTs exist across organs (e.g., macrophages), most CTs are highly specialized to deliver well-defined organ-specific functions in ASs. To demonstrate that ASpop varies not only by organ but also by AS, four heatmaps were made (one per CTann tools plus sc-proteomics, see Fig. C1 and Fig. C2); due to their scale and density, they are available in high-resolution on the companion website at cns-iu.github.io/hra-cell-type-populations-supporting-information#figures. Each heatmap lists CT labels on the x-axis and organ plus AS labels on the y-axis. Table field color represents the scaled mean value, i.e., z-score, see equation (1) for the percentage of CTs identified in each AS.

For each heatmap, data from a CTann tool was selected and processed to calculate the average CT percentage associated with all the ASs in an organ. The results were transformed from a data frame into a matrix (CTs by organ plus AS, concatenated into a combined label), where each matrix cell represents the average CT percentage measured for all CT and AS dyads. Finally, matrix values were converted to a standardized z-score, which was calculated using the formula in equation (1).

1 $$Z=\frac{(x-\mu )\,}{\sigma }$$

#### Computation of standardized z-score for CTs across ASs

Equation (1) shows *x* as an average CT percentage measure, where *μ* is the mean average CT percentage, and *σ* is the standard deviation mean average CT percentage. The z-score identifies how many standard deviations a data point is from the average mean. If the z-score is 0, values are close to the variable’s average; a z-score of 1 indicates that CT percentage values are 1 standard deviation higher than the mean for that CT, values of 2 are 2 standard deviations from the mean, etc.

Differences across organs and CTann become visible. For example, heart, lung, kidney, and pancreas, when annotated with Azimuth (see Fig. C1A), show distinct bands of CTs with a z-score of ~1.5, some up to 5. We observe similar patterns for the heart, liver, lung, pancreas, skin, and small/large intestines in CellTypist (see Fig. C1B), as well as breast, heart, liver, lung, pancreas, prostate, skin, small/large intestines, spleen, thymus, urinary bladder, and ureter for popV (see Fig. C1.C). High-resolution versions of the heatmaps and the code to generate them are listed on the companion website at cns-iu.github.io/hra-cell-type-populations-supporting-information#z-scores-for-cts-per-organ-and-as.

#### Datasets per AS

**Fig. 6:**
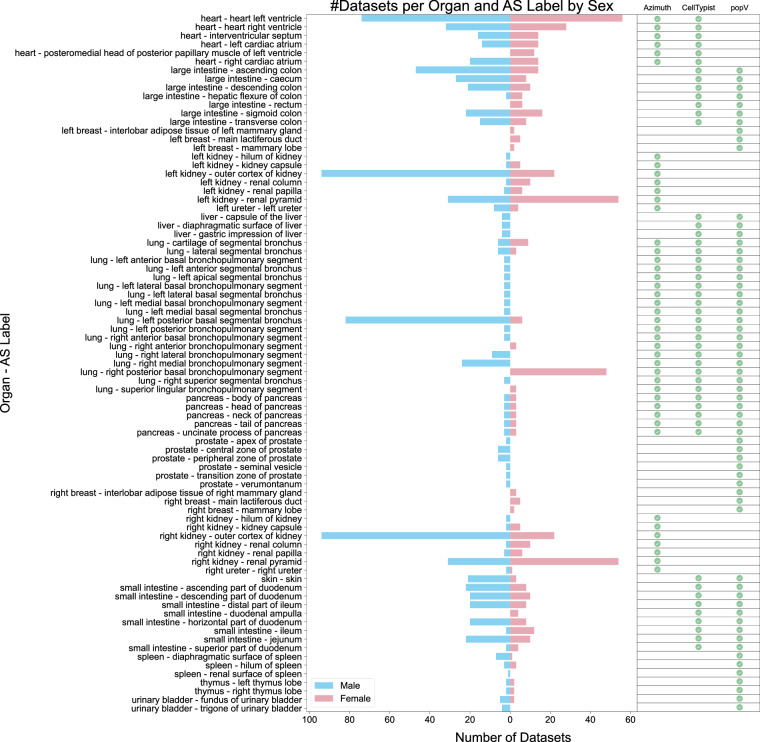
HRApop Atlas Datasets per sex-specific organ and AS. The number of HRApop Atlas Data per organ and AS combination (y-axis) is plotted on the x-axis, separated and colored by sex, with the CTann tool availability for the organ marked on the very right.

The population pyramid in Fig. 6 shows the coverage of HRApop Atlas Data across ASs and organs. Depicted is the number of datasets per organ and AS label by sex. Overlaps and gaps in coverage between CTann tools (see matrix on the right), across all organs and ASs, and between sex (color) become visible. The AS with the most registered HRApop Atlas Data is the outer cortex of the kidney (purl.obolibrary.org/obo/UBERON_0002189) for male, with 94 datasets run through Azimuth. Sc-proteomics datasets are omitted from this figure.

## Usage Notes

The **Data Records** section points to download links for 558 H5AD files for sc-transcriptomics data, CT populations with expert-provided CTann for sc-proteomics data, and HRAop data products. This section details two additional ways of accessing HRApop Atlas Data.

### Getting HRApop data via API queries

A Jupyter Notebook detailing programmatic access to CT populations for ASs, extraction sites, and datasets via grlc.io is available at cns-iu.github.io/hra-cell-type-populations-supporting-information#accessing-hrapop-data-via-hra-api.

### Visualizing CT populations for ASs, extraction sites, and datasets

**Fig. 7:**
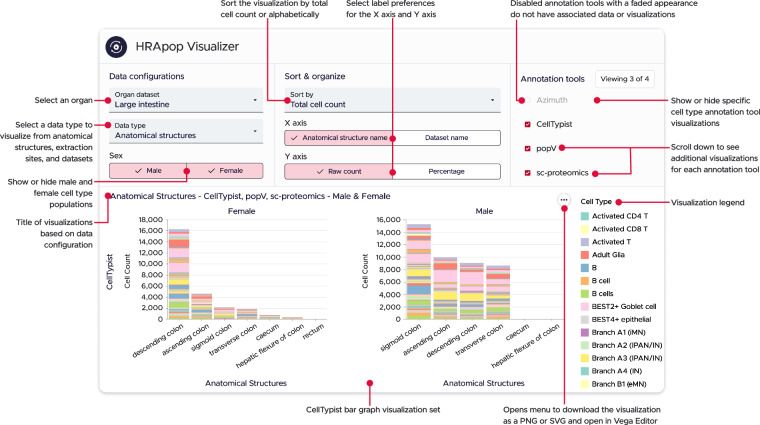
The HRApop Visualizer represents CT populations for ASs, extraction sites, and datasets as stacked bar graphs. As an example of an organ with CT populations from multiple CTann tools, the large intestine is shown, for which there are CT populations from CellTypist, popV, and sc-proteomics. In this screenshot, only the CT populations from CellTypist are shown (those from popV and sc-proteomics data are present on but cropped out). Note that the rectum (left) and caecum/hepatic flexure of colon (right) do indeed have bars, but the counts are so small that they are not rendered. Switching the y-axis to percentage makes these small counts visible as it changes the stacked bar graphs to 100% stacked bar graphs.

A web interface to inspect HRApop Atlas CT populations via stacked bar graphs, entitled “HRApop Visualizer,” is available at apps.humanatlas.io/hra-pop-visualizer. A tutorial is provided at cns-iu.github.io/hra-cell-type-populations-supporting-information/#visualizing-cell-type-populations-for-as-es-and-datasets. An example screenshot is shown in Fig. 7.

## Supplementary information

Supplementary Information

## Data Availability

Six major HRApop data products are available for download on Zenodo at 10.5281/zenodo.15603820. ASpop, DESpop, and corridors are also available via the HRA KG at purl.humanatlas.io/graph/hra-pop/v1.0 and on GitHub^114^, as well as in the form of canned SPARQL queries at apps.humanatlas.io/api/grlc/hra-pop.html. Links to all data are provided in Table S1, as is a complete listing of all code to construct and use HRApop. Examples for usage of this HRApop Atlas Data are available on the companion website at cns-iu.github.io/hra-cell-type-populations-supporting-information#usage-examples and the **Usage Notes** section.

## Code availability

The release code for HRApop v1.0 was archived on Zenodo for the DCTA Workflow^124^, the HRApop CTann Tool Containers^125^, and the RUI2CTpop Workflow^126^.

An overview of all GitHub repositories used to construct and use HRApop is provided in Table S1, separated by Major Data Products, Code (Construction), Code (Support), Collision Detection and Corridors, and Coverage and Visualization. All HRA UIs that expose/use HRApop are listed in Table S2. A companion website for this paper is available at cns-iu.github.io/hra-cell-type-populations-supporting-information and hosted in a GitHub repository^133^.

The Sankey diagram from Fig. 2 can be explored interactively at cns-iu.github.io/hra-cell-type-populations-supporting-information/sankey_universe_plotly.html. A version showing only HRApop Atlas Data is shown in Fig. S2, with an interactive version available at cns-iu.github.io/hra-cell-type-populations-supporting-information/sankey_atlas_plotly.html.
