# Supplementary material for: Cell Type Populations for 3D Anatomical Structures of the Human Reference Atlas
Source: Sci Data. 2026 Mar 19;13:716. doi: 10.1038/s41597-026-06642-4 (PMC13153392; doi:10.1038/s41597-026-06642-4)
Supplement: Supplementary file 1 — Supplementary Information [file 41597_2026_6642_MOESM1_ESM.pdf]

Supplementary Information

Cell Type Populations for 3D Anatomical Structures of the Human Reference Atlas

Andreas Bueckle[1*], Bruce W. Herr II[1*], Lu Chen[2], Daniel Bolin[1], Danial Qaurooni[1], Michael Ginda[1], Yashvardhan Jain[1], Aleix Puig-Barbe[3], Kristin Ardlie[4], Fusheng Wang[2,5], Katy Börner[1*]

[1] Department of Intelligent Systems Engineering, Luddy School of Informatics, Computing, and Engineering, Indiana University, Bloomington, IN, 47408, USA
[2] Department of Computer Science, Stony Brook University, Stony Brook, NY, 11794, USA
[3] European Molecular Biology Laboratory-European Bioinformatics Institute, Wellcome Genome Campus, Hinxton, Cambridge CB10 1SD, UK
[4] Broad Institute, Cambridge, MA, 02142, USA
[5] Department of Biomedical Informatics, Stony Brook University, Stony Brook, NY, 11794, USA

* Corresponding authors
abueckle@iu.edu
bherr@iu.edu
katy@iu.edu

Outlook

To improve coverage and quality of HRApop, the following steps are planned:

Long-term sustainability: The HRA and, by extension, HRApop is currently funded via the HuBMAP, SenNet, KPMP, Common Fund Data Ecosystem (CFDE), GTEx, and NIDDK. Funding that has been acquired since HRApop v1.0 was released comes via the Whole Person Reference Physiome Research and Coordination Center (WPP, 1U24AT013504-01), the Canadian Institute for Advanced Research (CIFAR) MacMillan Multiscale Human (cifar.ca/research-programs/cifar-macmillan-multiscale-human), and the Stiftung Charité via Berlin Institute of Health at Charité (BIH). This funding supports regular HRApop releases, with data products made available via the HRA Portal, Zenodo[1], and the HRA KG[2] (lod.humanatlas.io/graph/hra-pop).

Increase number of RUI registered datasets: As of March 2026, the HuBMAP and SenNet portals list 900 and 227 datasets with 3D extraction sites that are currently in QA/QC status but will be published soon. Outreach efforts to authors of peer-reviewed, published papers are ongoing to register their data for use in the DCTA Workflow. The HRApop effort will also integrate data from Tabula Sapiens[3], KPMP, the Helmsley Gut Cell Atlas[4], and the Deeply Integrated human Single-Cell Omics (DISCO) database[5], which has a total of 21,330 datasets, out of which 32.8% of the total data is from a healthy human body, across 166 unique ASs. Other potential sources for high-quality datasets have been captured in Hemberg et al.'s recent article on large cell atlases[6].

Scale up tissue registration via millitomes: A millitome[7] (from Latin *mille*, meaning "thousand," and the Greek *temnein*, meaning "to cut") is a device designed to hold a freshly procured organ and facilitate cutting it into many small tissue blocks of well defined size for usage in sc-analysis and HRA construction. It is used to produce uniformly sized slices or cubes of tissue material that can be registered to 3D reference objects. Using a millitome improves efficiency by enabling consistent, high-throughput sampling. Recently, 727 HuBMAP tissue blocks, containing 33988239 cells from kidney, pancreas, and reproductive system tissue, were

registered via the millitome process. They will be included in the next HRApop run, as part of the 10th HRA release.

Improve generalizability: The 104 sc-proteomics datasets in HRApop v1.0 were presented as a generalization from sc-transcriptomics datasets. In the future, and in synergy with HRA Vasculature Common Coordinate Framework (VCCF) construction efforts[8,9] around endothelial cell environments[10], more CODEX[11] datasets and new modalities, such as the Spatial Multiomics Single-Cell Imaging platform CosMx[12], will be added to HRApop. A recent paper[10] analyzed 399 spatially resolved omics datasets from 14 studies comprising 12 tissue types and a total of 47,349,496 segmented and annotated cells.

Increase number of CTann tools used to enable more benchmarking: Currently, HRApop uses three well-established CTann tools backed by scientific publications describing the methods, results, and validations for each tool. Results are presented as CT populations by CTann tool—users can pick their favorite tool and data or perform comparisons and benchmarks between CTann tools[13]. Future HRA releases will feature additional CTann tools such as FR-Match[14] and Pan-Human Azimuth (satijalab.org/pan_human_azimuth) in support of improved cell type annotation, CTann tool comparisons, and benchmarking.

Add biomarker sets: For HRApop v1.0, the top biomarkers per CT per dataset were computed using *scanpy*'s *rank_gene_groups()* method (scanpy.readthedocs.io/en/stable/generated/scanpy.tl.rank_genes_groups.html). In future HRApop releases, additional sets of top biomarkers will be provided to the user by running, e.g., NS-Forest[15] during the annotation phase of the DCTA Workflow.

Decrease run time for HRApop code: For HRApop v1.0, the DCTA Workflow started on Thu, May 15, 2025, ran for about 10 days, and finished on Sunday, May 25, 2025. It averaged 87.63 dataset annotation runs per hour. Annotations took about 8.59 days to finish. This long runtime is primarily due to the complexity of the annotation pipeline, including annotation, crosswalking, and mean gene expression per cell type, to cover over 10,000 datasets and about ten million cells. Targeted optimization of the algorithms and workflows combined with more hardware resources and re-using annotations from prior runs will be required to reduce runtime. Work is underway to save annotations between runs to skip the re-annotation step. After a 22-day QA phase, the RUI2CTpop Workflow started on June 16 at 5:55:27 PM EDT and finished about four hours later at 10:07:11 PM EDT the same day. A full log is linked in **Table S1**. In the future, the run time for the DCTA Workflow will be decreased by using high performance computing (HPC), e.g., Big Red at Indiana University (kb.iu.edu/d/brcc). Also, the crosswalking will be moved to the RUI2CTpop Workflow, which will decrease runtime and increase the modularity of both workflows.

Supplementary Figures

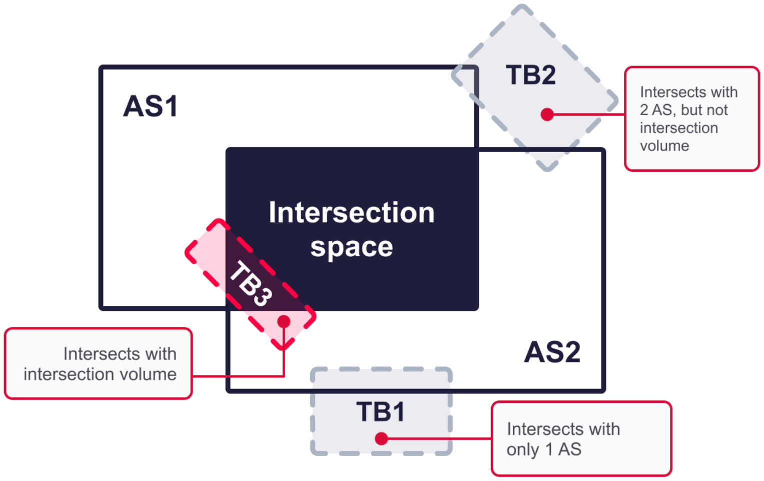

Fig. S1. **Intersection space and intersection volumes.** Two intersecting ASs create a 3D intersection space. Three exemplary tissue blocks intersect with one AS (TB1), both ASs (TB2), or both and their intersection space (TB3).

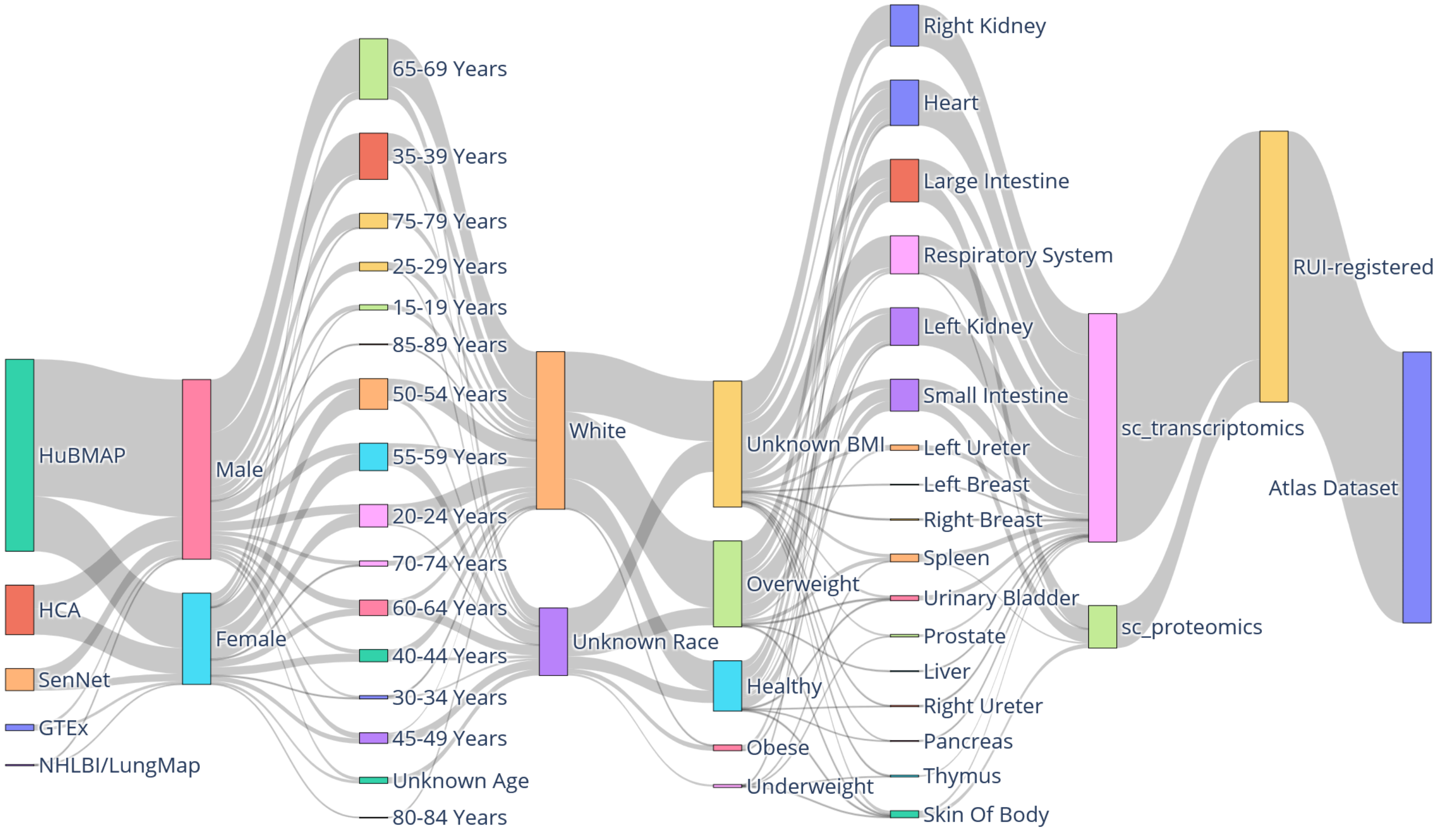

Fig. S2. Sankey Diagram for HRApop Atlas Data. Interactive data visualization can be explored at cns-iu.github.io/hra-cell-type-populations-supporting-information/sankey_atlas_plotly.html.

Supplementary Tables

Table S1. Listing of all GitHub repositories used to construct and use HRApop.

Name	Description	URL
**Major Data Products**		
DESpop	Provided in a variety of formats (including JSON-LD and Terse RDF Triple Language [Turtle][16]) via the HRA KG and as JSON-LD on GitHub	Zenodo[1]<br><br>GitHub (JSON-LD):<br>github.com/x-atlas-consortia/hra-pop/blob/main/output-data/v1.0/atlas-enriched-dataset-graph.jsonld<br><br>For datasets on GitHub (CSV):<br>github.com/x-atlas-consortia/hra-pop/blob/main/output-data/v1.0/reports/atlas-ad-hoc/cell-types-per-dataset.csv<br><br>For datasets on grlc.io (canned SPARQL query):<br>apps.humanatlas.io/api/grlc/hra-pop.html#get-/cell-types-per-dataset<br><br>For extraction sites on GitHub (CSV):<br>github.com/x-atlas-consortia/hra-pop/blob/main/output-data/v1.0/reports/atlas-ad-hoc/cell-types-per-extraction-site.csv<br><br>For extraction sites on grlc.io (canned SPARQL query):<br>apps.humanatlas.io/api/grlc/hra-pop.html#get-/cell-types-per-extraction-site<br><br>HRA KG:<br>cdn.humanatlas.io/digital-objects/graph/hra-pop/latest/assets/atlas-enriched-dataset-graph.jsonld
ASpop	Provided via the HRA KG and via GitHub	Zenodo[1]<br><br>HRA KG:<br>cdn.humanatlas.io/digital-objects/graph/hra-pop/v1.0/assets/atlas-as-cell-summaries.jsonld<br><br>GitHub (JSON-LD):<br>github.com/x-atlas-consortia/hra-pop/blob/main/output-data/v1.0/atlas-as-cell-summaries.jsonld<br><br>GitHub (CSV):<br>github.com/x-atlas-consortia/hra-pop/blob/main/output-data/v1.0/reports/atlas-ad-hoc/cell-types-in-anatomical-structurescts-per-as.csv<br><br>grlc.io (canned SPARQL query):<br>apps.humanatlas.io/api/grlc/hra-pop.html#get-/cell_types_in_anatomical_structurescts_per_as
Cell instances	Cell instances for sc-transcriptomics datasets in HRApop v1.0	Zenodo[1]

Cell instances with top-10,000 biomarkers	Cell instances for sc-transcriptomics datasets in HRApop v1.0 with top-10,000 biomarkers	Zenodo[1]
Corridors	Corridors for extraction sites for HRApop v1.0	Zenodo[1]<br><br>GitHub: github.com/x-atlas-consortia/hra-pop/blob/main/output-data/v1.0/atlas-as-cell-summaries.jsonld<br><br>HRA KG: lod.humanatlas.io/graph/hra-pop/v1.0/
HRApop quality control	ZIP folder with QC metrics for all 558 sc-transcriptomics datasets used in the DCTA Workflow	Zenodo[1]
**Code (Construction)**		
Input data for DCTA and RUI2CTpop Workflows	Contains H5AD files (DCTA) and cell type populations for datasets and metadata (RUI2CTpop)	Globus[17]
DCTA Workflow	A set of scripts to download H5AD files, execute the Docker containers in HRApop CTann Tool Containers, and output CT populations and data metadata as input for the RUI2CTpop Workflow.	Release for HRApop v1.0: Zenodo[18]<br>Release for HRApop v1.0: GitHub[19]<br>Active repository: GitHub[20]
HRApop CTann Tool Containers	Docker containers for running CTann tools over H5AD files.	Release for HRApop v1.0: Zenodo[21]<br>Release for HRApop v1.0: GitHub[22]<br>Active repository: GitHub[23]
RUI2CTpop Workflow	A collection of scripts to compile HRApop from the output of the DCTA Workflow, which first provides CT populations and dataset metadata, then copies those files over to GitHub[24,25]. Scripts running over these input files are also on GitHub[26]. Output data is provided at github.com/x-atlas-consortia/hra-pop/tree/main/output-data/v1.0. For downstream	Release for HRApop v1.0: Zenodo[28]<br>Release for HRApop v1.0: GitHub[29]<br>Active repository: GitHub[25]

	analysis, reports based on SPARQL queries are on GitHub[27].<br><br>Full log is available at raw.githubusercontent.com/x-atlas-consortia/hra-pop/refs/heads/main/output-data/v1.0/log.txt.<br><br>File to capture the creation date of the pipeline having finished is at github.com/x-atlas-consortia/hra-pop/blob/main/output-data/v1.0/CREATION_DATE.<br><br>Readme for the most recent run is present at github.com/x-atlas-consortia/hra-pop/blob/main/output-data/v1.0/README.md.	
Dataset info	Dataset IDs for all 16,293 datasets originally downloaded, incl. donor sex, assay type, CTann tool run, and unique CTs identified	GitHub[30]
CTann crosswalks	A collection of CSV files that link CT labels from Azimuth, CellTypist, and popV to CL or PCL IDs so they can be connected to the ASCT+B tables and other HRA Digital Objects. For example, the most recent crosswalk CSV file for Azimuth is available for download at the bottom of the HRA Digital Object landing page at lod.humanatlas.io/ctann/azimuth/latest.	lod.humanatlas.io/ctann
CTs level mapping	Report and queries to map crosswalked, unique CTs to higher-level CTs	Report:<br>github.com/x-atlas-consortia/hra-pop/blob/main/output-data/v1.0/reports/atlas-ad-hoc/cell-types-level-mapping.csv<br><br>Query:<br>github.com/x-atlas-consortia/hra-pop/blob/main/queries/atlas-ad-hoc/cell-types-level-mapping.rq

		Report (long): github.com/x-atlas-consortia/hra-pop/blob/main/output-data/v1.0/reports/atlas-ad-hoc/cell-types-level-mapping-long.csv<br><br>Query: github.com/x-atlas-consortia/hra-pop/blob/main/queries/atlas-ad-hoc/cell-types-level-mapping-long.rq
Not crosswalked	Reports for cell IDs in proteomics data that were not crosswalked during the DCTA Workflow	Input for RUI2CTpop Workflow: GitHub[31]<br><br>HRApop Atlas: GitHub[32]
CT populations and metadata for sc-transcriptomics and sc-proteomics data, produced by the DCTA Workflow and serving as input for the RUI2CTpop Workflow	**sc-transcriptomics-cell-summaries.jsonld.gz**: Contains CT populations for all sc-transcriptomics datasets annotated with a CTann tool in JSON-LD.<br><br>**sc-transcriptomics-dataset-metadata.csv**: Contains organ, donor metadata. It also features a handler ID to denote which portal a dataset was downloaded from, a UUID, an assay type, and tissue provider information.<br><br>**sc-proteomics-cell-summaries.jsonld:** Contains CT populations for all sc-proteomics datasets in JSON-LD. The summaries are produced via the DCTA Workflow using the code at github.com/cns-iu/hra-node-dist-vis/blob/main/scripts/build-cell-summaries.js, which queries a listing of all possible sc-proteomics datasets (see GitHub[33]).<br><br>**sc-transcriptomics-dataset-metadata.csv:** Contains donor, tissue block, and data IDs for sc-proteomics datasets.	GitHub (all)[24]<br><br>GitHub (cell summaries for sc-protemics only)[34]
**Code (Support)**		
HRA Registrations	Manually curated HRA	GitHub[35]

	Dataset Graphs. This repository holds static dataset graphs for use in the EUI and other HRA applications. All registration Digital Objects are published to hubmapconsortium.github.io/hra-registrations/. All registrations are at hubmapconsortium.github.io/hra-registrations/**name-in-root-folder**/rui_locations.jsonld	
HRA Registrations Processor	Command line interface to simplify creating rui_locations.jsonld files	github.com/hubmapconsortium/hra-rui-locations-processor
Querying HRApop	A collection of SPARQL queries for use in this paper and for downstream analysis (to identify counts and get CT populations, biomarkers)	grlc.io/api-git/hubmapconsortium/ccf-grlc/subdir/hra-pop
RUI	Stand-alone RUI use to register tissue blocks	Stand-alone RUI: apps.humanatlas.io/rui<br><br>RUI code repository: github.com/hubmapconsortium/hra-ui/tree/main/apps/ccf-rui
EUI	Deployed stand-alone RUI extraction site sets displayed in the EUI	hubmapconsortium.github.io/hra-registrations
CTann tools	Azimuth: v0.4.6<br>CellTypist: v1.6<br>popV: github.com/YosefLab/popV/tree/2d29c9a290d2015ec65ef0ef9f0e6b6d2277e7bb	Azimuth: azimuth.hubmapconsortium.org<br>CellTypist: www.celltypist.org<br>popV: github.com/YosefLab/PopV
**Collision Detection and Corridors**		
HRA Mesh Collision API	Given an extraction site, get mesh-based collisions with the 3D reference object.	Code: github.com/hubmapconsortium/hra-tissue-block-annotation<br><br>Deployed: pfn8zf2gtu.us-east-2.awsapprunner.com/get-collisions<br><br>Public endpoint: apps.humanatlas.io/api/v1/collisions<br><br>Documentation for endpoint: apps.humanatlas.io/api/#post-/v1/collisions<br><br>Example result:

		github.com/hubmapconsortium/hra-tissue-block-annotation/blob/main/examples/test-registration-collisions.json
3D Corridor Generation API	Given an extraction site, generate a corridor with the 3D reference object as a GLB file	Code: github.com/hubmapconsortium/hra-corridor-generation<br><br>Deployed: dwwcpwad72.us-east-2.awsapprunner.com/get-corridor<br><br>Public endpoint: apps.humanatlas.io/api/v1/corridor<br><br>Documentation for endpoint: apps.humanatlas.io/api/#post-/v1/corridor
Corridors for downloading	Contains all corridor GLB files	github.com/x-atlas-consortia/hra-pop/tree/main/output-data/v1.0/corridors
Mesh-mesh collision and annotation		Code: github.com/hubmapconsortium/hra-glb-mesh-collisions<br><br>On PyPi: pypi.org/project/hra-glb-mesh-collisions/0.1.0/
**Coverage and Visualization**		
CT Populations by AS	Provides a query for an overview of all AS-CT combinations in ASpop of HRApop v1.0, with sex, tool, CT, and cell percentage	apps.humanatlas.io/api/grlc/hra-pop.html#get-/cell_types_in_anatomical_structurescts_per_as.
Supporting Information for this paper	Contains assets for the companion website at cns-iu.github.io/hra-cell-type-populations-supporting-information/	github.com/cns-iu/hra-cell-type-populations-supporting-information
Sankey Diagram for HRApop Atlas	Contains the deployed Sankey diagram for HRApop Atlas	cns-iu.github.io/hra-cell-type-populations-supporting-information/sankey_atlas_plotly.html
Sankey Diagram for input for RUI2CTpop Workflow	Contains the deployed Sankey diagram for the input for RUI2CTpop Workflow	cns-iu.github.io/hra-cell-type-populations-supporting-information/sankey_universe_plotly.html
HRApop Counts	Contains numbers reported in this paper	github.com/cns-iu/hra-cell-type-populations-supporting-information/blob/main/counts/hra_pop_counts.ipynb

Table S2. Listing of all HRA applications that use HRApop data.

Name	Description	URL[36]
FTU Explorer	Web-deployed UI to view and explore cell type populations from experimental datasets for 2D FTU illustrations	apps.humanatlas.io/ftu-explorer
HRA Organ Gallery[37,38]	Enables an immersive view of the HRA by showing 71 reference organs and 1,100+ tissue blocks alongside CT populations in VR[37,38].	humanatlas.io/hra-organ-gallery
HRA API	Provides programmatic access to the HRA, enabling integration with other applications and services. A SPARQL endpoint for the HRA API allows users to write their own SPARQL queries. Canned queries for HRApop are available at apps.humanatlas.io/api/grlc/hra-pop.html.	API documentation: apps.humanatlas.io/api<br><br>The HRA API on the HRA Portal: humanatlas.io/api
HRApop Visualizer	Web-deployed UI to visualize CT populations for 73 ASs, 230 extraction sites, and 662 HRApop Atlas datasets using stacked bar graphs, faceted by sex and CTann tool	apps.humanatlas.io/hra-pop-visualizer

Table S3. CTann settings. Docker containers with full contexts for the three CTann tools are available on GitHub[39].

Tool	Version	Code base	Models	Requirements
Azimuth	v0.4.6	R	kidneyref/Kidney_L3/annotation.l3<br>lungref/Lung_v2_finest_level/ann_finest_level<br>heartref/Heart_L2/celltype.l2<br>humancortexref/subclass<br>pancreasref/Pancreas_L1/annotation.l1<br>pbmcref/Human_PBMC_L2/celltype.l2<br>bonemarrowref/Bone_marrow_L2/celltype.l2<br>adiposeref/Adipose_L2/celltype.l2	anndata==0.9.1<br>contourpy==1.0.7<br>cycler==0.11.0<br>fonttools==4.39.4<br>h5py==3.8.0<br>importlib-metadata==6.6.0<br>importlib-resources==5.12.0<br>joblib==1.2.0<br>kiwisolver==1.4.4<br>llvmlite==0.40.1rc1<br>matplotlib==3.7.1<br>natsort==8.3.1<br>networkx==3.1<br>numba==0.57.0<br>numpy==1.24.3<br>packaging==23.1<br>pandas==2.0.2<br>patsy==0.5.3<br>Pillow==9.5.0<br>pynndescent==0.5.10<br>pyparsing==3.0.9<br>python-dateutil==2.8.2<br>pytz==2023.3<br>scanpy==1.9.3<br>scikit-learn==1.2.2<br>scipy==1.10.1<br>seaborn==0.12.2<br>session-info==1.0.0<br>six==1.16.0<br>statsmodels==0.14.0<br>stdlib-list==0.8.0<br>threadpoolctl==3.1.0<br>tqdm==4.65.0<br>tzdata==2023.3<br>umap-learn==0.5.3<br>zipp==3.15.0
CellTypist	v1.6	Python	Human_Lung_Atlas.pkl<br>Alternative: Cells_Lung_Airway.pkl<br>Adult_Human_Skin.pkl<br>Adult_Human_PancreaticIslet.pkl<br>Adult_Human_PancreaticIslet.pkl<br>Healthy_Adult_Heart.pkl<br>Healthy_Human_Liver.pkl<br>Human_AdultAged_Hippocampus.pkl<br>Human_Longitudinal_Hippocampus.pkl<br>Cells_Intestinal_Tract.pkl	anndata==0.9.2<br>celltypist==1.6.1<br>certifi==2023.7.22<br>charset-normalizer==3.3.1<br>click==8.1.7<br>contourpy==1.1.1<br>cycler==0.12.1<br>et-xmlfile==1.1.0<br>fonttools==4.43.1<br>h5py==3.10.0<br>idna==3.4<br>igraph==0.10.8<br>joblib==1.3.2<br>kiwisolver==1.4.5<br>leidenalg==0.10.1<br>llvmlite==0.41.1<br>matplotlib==3.8.0<br>natsort==8.4.0

				networkx==3.2<br>numba==0.58.1<br>numpy==1.24.4<br>openpyxl==3.1.2<br>packaging==23.2<br>pandas==2.0.3<br>patsy==0.5.3<br>Pillow==10.1.0<br>pynndescent==0.5.10<br>pyparsing==3.1.1<br>python-dateutil==2.8.2<br>pytz==2023.3.post1<br>requests==2.31.0<br>scanpy==1.9.5<br>scikit-learn==1.3.2<br>scipy==1.11.3<br>seaborn==0.13.0<br>session-info==1.0.0<br>six==1.16.0<br>statsmodels==0.14.0<br>stdlib-list==0.9.0<br>texttable==1.7.0<br>threadpoolctl==3.2.0<br>tqdm==4.66.1<br>tzdata==2023.3<br>umap-learn==0.5.4<br>urllib3==2.0.7
popV	See commit	Python	model: Bladder<br>organ_level:urinary bladder<br><br>model: Blood<br>organ_level: blood<br><br>model: Bone_Marrow<br>organ_level: bone marrow<br><br>model: Eye<br>organ_level: eye<br><br>model: Fat<br>organ_level: adipose tissue<br><br>model: Large_Intestine<br>organ_level: large intestine<br><br>model: Liver<br>organ_level: liver<br><br>model: Lung<br>organ_level: lung<br><br>model: Lymph_Node<br>organ_level: mesenteric lymph node<br><br>model: Mammary<br>organ_level: mammary gland<br><br>model: Pancreas<br>organ_level: pancreas	absl-py==2.1.0<br>aiohappyeyeballs==2.4.0<br>aiohttp==3.10.5<br>aiosignal==1.3.1<br>anndata==0.10.9<br>annoy==1.17.3<br>array_api_compat==1.8<br>astunparse==1.6.3<br>attrs==24.2.0<br>bbknn==1.6.0<br>beautifulsoup4==4.12.3<br>celltypist==1.6.3<br>certifi==2024.8.30<br>charset-normalizer==3.3.2<br>chex==0.1.86<br>click==8.1.7<br>contextlib2==21.6.0<br>contourpy==1.3.0<br>cycler==0.12.1<br>Cython==3.0.11<br>docrep==0.3.2<br>et-xmlfile==1.1.0<br>etils==1.9.4<br>fbpca==1.0<br>filelock==3.15.4<br>flatbuffers==24.3.25<br>flax==0.9.0<br>fonttools==4.53.1<br>frozenlist==1.4.1<br>fsspec==2024.9.0<br>gast==0.6.0<br>gdown==5.2.0

			model: Prostate<br>Organ_level: prostate gland<br><br>model: Salivary Gland<br><br>model: Skin<br>Organ_level: skin<br><br>model: Small_Intestine<br>organ_level: small intestine<br><br>model: Spleen<br>organ_level: spleen<br><br>model: Thymus<br>organ_level: thymus<br><br>model: Tongue<br><br>model: Trachea<br>organ_level: trachea<br><br>model: Uterus<br>organ_level: uterus<br><br>model: Vasculature<br>organ_level: blood vasculature	geosketch==1.2<br>google-pasta==0.2.0<br>grpcio==1.66.1<br>h5py==3.11.0<br>harmony-pytorch==0.1.8<br>huggingface-hub==0.24.6<br>humanize==4.10.0<br>idna==3.8<br>igraph==0.11.6<br>importlib_resources==6.4.4<br>intervaltree==3.1.0<br>jax==0.4.31<br>jaxlib==0.4.31<br>Jinja2==3.1.4<br>joblib==1.4.2<br>keras==3.5.0<br>kiwisolver==1.4.7<br>legacy-api-wrap==1.4<br>leidenalg==0.10.2<br>libclang==18.1.1<br>lightning==2.1.4<br>lightning-utilities==0.11.7<br>llvmlite==0.43.0<br>Markdown==3.7<br>markdown-it-py==3.0.0<br>MarkupSafe==2.1.5<br>matplotlib==3.9.2<br>mdurl==0.1.2<br>ml-dtypes==0.4.0<br>ml_collections==0.1.1<br>mpmath==1.3.0<br>msgpack==1.0.8<br>mudata==0.3.1<br>multidict==6.0.5<br>multipledispatch==1.0.0<br>namex==0.0.8<br>natsort==8.4.0<br>nest-asyncio==1.6.0<br>networkx==3.3<br>numba==0.60.0<br>numpy==1.26.4<br>numpyro==0.15.2<br>nvidia-cublas-cu12==12.1.3.1<br>nvidia-cuda-cupti-cu12==12.1.105<br>nvidia-cuda-nvrtc-cu12==12.1.105<br>nvidia-cuda-runtime-cu12==12.1.10<br>5<br>nvidia-cudnn-cu12==9.1.0.70<br>nvidia-cufft-cu12==11.0.2.54<br>nvidia-curand-cu12==10.3.2.106<br>nvidia-cusolver-cu12==11.4.5.107<br>nvidia-cusparse-cu12==12.1.0.106<br>nvidia-nccl-cu12==2.20.5<br>nvidia-nvjitlink-cu12==12.6.68<br>nvidia-nvtx-cu12==12.1.105<br>obonet==1.1.0<br>OnClass==1.3<br>openpyxl==3.1.5<br>opt-einsum==3.3.0<br>optax==0.2.3

				optree==0.12.1<br>orbax-checkpoint==0.6.1<br>packaging==24.1<br>pandas==1.5.3<br>patsy==0.5.6<br>pillow==10.4.0<br>PopV @ git+github.com/czbiohub/PopV@2d29c9a290d2015ec65ef0ef9f0e6b6d2277e7bb<br>protobuf==4.25.4<br>psutil==6.0.0<br>Pygments==2.18.0<br>pynndescent==0.5.13<br>pyparsing==3.1.4<br>pyro-api==0.1.2<br>pyro-ppl==1.9.1<br>PySocks==1.7.1<br>python-dateutil==2.9.0.post0<br>pytorch-lightning==2.4.0<br>pytz==2024.1<br>PyYAML==6.0.2<br>regex==2024.7.24<br>requests==2.32.3<br>rich==13.8.0<br>safetensors==0.4.5<br>scanorama==1.7.4<br>scanpy==1.10.2<br>scikit-learn==1.1.3<br>scikit-misc==0.5.1<br>scipy==1.14.1<br>scvi-tools==1.1.6<br>seaborn==0.13.2<br>sentence-transformers==3.0.1<br>session_info==1.0.0<br>six==1.16.0<br>sortedcontainers==2.4.0<br>soupsieve==2.6<br>statsmodels==0.14.2<br>stdlib-list==0.10.0<br>sympy==1.13.2<br>tensorboard==2.17.1<br>tensorboard-data-server==0.7.2<br>tensorflow==2.17.0<br>tensorflow-io-gcs-filesystem==0.37.1<br>tensorstore==0.1.65<br>termcolor==2.4.0<br>texttable==1.7.0<br>threadpoolctl==3.5.0<br>tokenizers==0.19.1<br>toolz==0.12.1<br>torch==2.4.1<br>torchmetrics==1.4.1<br>tqdm==4.66.5<br>transformers==4.44.2<br>triton==3.0.0<br>typing_extensions==4.12.2<br>umap-learn==0.5.6<br>urllib3==2.2.2

				Werkzeug==3.0.4<br>wget==3.2<br>wrapt==1.16.0<br>yarl==1.9.11<br>zenodo-get==1.5.1<br>zipp==3.20.1

Table S4. Reports about dataset and cell counts from the RUI2CTpop Workflow.

**Counts**		
Input for RUI2CTpop Workflow: sc-transcriptomics cell counts	Contains the number of annotated cells in sc-transcriptomics datasets that are part of the input for RUI2CTpop Workflow	github.com/x-atlas-consortia/hra-pop/blob/main/output-data/v1.0/reports/universe-ad-hoc/universe-sc-transcriptomics-cell-counts.csv
Input for RUI2CTpop Workflow: sc-transcriptomics cell counts (with pre-annotated cell counts)	Contains the number of annotated cells in sc-transcriptomics datasets that are part of the input for RUI2CTpop Workflow that have been mapped via CTann tools (see **Box 1**). Pre-annotated cell counts are the number of rows in the cell by gene matrix of the H5AD file. Some cells may not get annotated for various reasons, thus the discrepancy	github.com/x-atlas-consortia/hra-pop/blob/main/output-data/v1.0/reports/universe-ad-hoc/universe-sc-transcriptomics-cell-instance-counts.csv
Input for RUI2CTpop Workflow: sc-proteomics cell counts	Contains the number of annotated cells in sc-proteomics datasets that are part of the HRApop Atlas	github.com/x-atlas-consortia/hra-pop/blob/main/output-data/v1.0/reports/universe-ad-hoc/universe-sc-proteomics-cell-counts.csv
HRApop Atlas: sc-transcriptomics cell counts	Contains the number of annotated cells in sc-transcriptomics datasets that are part of the HRApop Atlas	github.com/x-atlas-consortia/hra-pop/blob/main/output-data/v1.0/reports/atlas-ad-hoc/atlas-sc-transcriptomics-cell-counts.csv
HRApop Atlas: sc-proteomics cell counts	Contains the number of annotated cells in sc-proteomics datasets that are part of the HRApop Atlas	github.com/x-atlas-consortia/hra-pop/blob/main/output-data/v1.0/reports/atlas-ad-hoc/atlas-sc-proteomics-cell-counts.csv
HRApop Atlas: Number of extraction sites per AS and intersection volume/percentage of the extraction site (see **Box 1**)	Lists extraction site, organ/AS, intersection volume, AS volume and tissue block volume.	github.com/x-atlas-consortia/hra-pop/blob/main/output-data/v1.0/reports/atlas/table-s5.csv
SPARQL queries	Contains SPARQL queries for all reports generated in in the output data folder on GitHub[40]	github.com/x-atlas-consortia/hra-pop/tree/main/queries

Table S5. CT choices. 26 of the 201 CTs were classified under two different high-level CTs. This table shows the decision process used to select the most appropriate grouping class for each CT.

CT	Chosen Category	Non-chosen category	Rationale
hematopoietic stem cell	hematopoietic cell	stem cell	Reflects lineage identity central to its biological role
neuroendocrine cell	epithelial cell	neural cell	Developmentally and structurally epithelial
pancreatic D cell	epithelial cell	neural cell	Developmentally and structurally epithelial
intestinal crypt stem cell of large intestine	stem cell	epithelial cell	Stemness prioritized as foundational to epithelial renewal
intestinal crypt stem cell of small intestine	stem cell	epithelial cell	Stemness prioritized as foundational to epithelial renewal
lung interstitial macrophage	hematopoietic cell	connective tissue cell	Lineage from hematopoietic system prioritized over location
pulmonary neuroendocrine cell	epithelial cell	neural cell	Developmentally and structurally epithelial
granulocyte monocyte progenitor cell	hematopoietic cell	bone cell	Reflects hematopoietic lineage over location in the bone marrow
mesenchymal stem cell of adipose tissue	stem cell	connective tissue cell	Stemness and multipotency prioritized over local connective tissue association.
mesenchymal stem cell of abdominal adipose tissue	stem cell	connective tissue cell	Stemness and multipotency prioritized over local connective tissue association.
central nervous system macrophage	hematopoietic cell	neural cell	Immune lineage prioritized over CNS location
vascular leptomeningeal cell	connective tissue cell	neural cell	Mesenchymal origin and structural connective function prioritized over CNS location
mature microglial cell	hematopoietic cell	neural cell	Immune lineage prioritized over CNS location
ependymal cell	neural cell	epithelial cell	Derived from neuroectoderm and part of CNS despite epithelial morphology
smooth muscle cell of the brain vasculature	muscular cell	neural cell	Reflects muscular identity, over location
mesenchymal stem cell	stem cell	connective tissue cell	Stemness and multipotency prioritized over local connective tissue association.
basal cell of epidermis	stem cell	epithelial cell	Stemness emphasized as central to epidermal renewal

choroid plexus epithelial cell	neural cell	epithelial cell	Derived from neuroectoderm and part of CNS despite epithelial morphology
microglial cell	hematopoietic cell	neural cell	Primitive macrophage origin prioritized over CNS-resident role
type D cell of colon	epithelial cell	neural cell	Developmentally and structurally epithelial
type EC enteroendocrine cell	epithelial cell	neural cell	Developmentally and structurally epithelial
cycling type EC enteroendocrine cell	epithelial cell	neural cell	Developmentally and structurally epithelial
type D cell of small intestine	epithelial cell	neural cell	Developmentally and structurally epithelial
retinal blood vessel endothelial cell	endothelial cell	neural cell	Endothelial identity and vascular role prioritized over retinal location
retinal pigment epithelial cell	epithelial cell	neural cell	Morphology and function as barrier epithelium prioritized over retinal location
endosteal cell	bone cell	epithelial cell	Skeletal lineage and role in bone homeostasis prioritized over lining morphology

Table S6. Mean and median ribosomal (ribo) and mitochondrial (mito) gene percentages across datasets per organ with standard deviations.

Organ name	Mean % ribo	Median % ribo	SD % ribo	Mean % mito	Median % mito	SD % mito
Heart	1.91031	0.795	2.29689	1.13329	0.751	1.10808
Large intestine	1.90185	1.06	1.68555	2.26706	1.416	3.06981
Left breast	5.608	5.608	0.23759	1.227	1.227	0.19233
Left kidney	2.70441	3.176	1.96414	7.07311	6.527	4.67822
Left ureter	5.29831	4.22	3.30931	3.551	1.771	3.61471
Liver	0.4425	0.4425	0.34814	4.368	4.368	0.86256
Male reproductive system	1.74633	0	2.84662	0.38367	0	0.59562
Pancreas	3.772	3.772	0.90922	1.796	1.796	0.55539
Respiratory system	1.4686	0.6845	2.85492	7.65423	4.27	10.99355
Right breast	0	0	0	0	0	0
Right kidney	3.18254	4.203	1.85188	7.2753	6.546	4.62628
Right ureter	4.947	4.142	1.3943	6.38167	7.423	1.80364
Skin of body	4.20729	4.583	3.01273	1.14514	1.382	0.8035
Small intestine	1.68298	1.2865	1.08863	1.72367	0.8055	2.3198
Spleen	8.41493	5.125	8.28201	9.10587	6.387	8.06477
Thymus	5.1735	4.917	0.97742	1.41225	1.3665	0.17922
Urinary bladder	2.9515	2.799	1.09684	1.82742	1.459	1.00274

Table S7. Mean number of genes with positive counts and mean total number of genes per organ.

Organ name	Mean number of genes with positive counts	Mean total number of genes
Heart	1154.92	1114.93
Large intestine	1702.50	2855.92
Left breast	3346.59	3387.70
Left kidney	1057.33	2077.37
Left ureter	1791.87	3225.29
Liver	1431.25	2491.35
Male reproductive system	1763.15	2469.97
Pancreas	3755.18	3327.74
Respiratory system	1344.93	3095.90
Right breast	658.07	1706.84
Right kidney	573.32	1063.03
Right ureter	1420.56	2583.12
Skin of body	2098.12	2344.72
Small intestine	2866.54	7428.50
Spleen	1479.40	2897.63
Thymus	3206.91	3403.37
Urinary bladder	1902.85	3257.01

References

1. Bueckle, A., Herr II, B. W. & Börner, K. HRApop v1.0. (2025). https://doi.org/10.5281/zenodo.15603820.
2. Bueckle, A. *et al.* Construction, Deployment, and Usage of the Human Reference Atlas Knowledge Graph. *Sci. Data* **12**, 1100 (2025). https://doi.org/10.1038/s41597-025-05183-6.
3. The Tabula Sapiens Consortium* *et al.* The Tabula Sapiens: A multiple-organ, single-cell transcriptomic atlas of humans. *Science* **376**, eabl4896 (2022). https://doi.org/10.1126/science.abl4896.
4. Zilbauer, M. *et al.* A Roadmap for the Human Gut Cell Atlas. *Nat. Rev. Gastroenterol. Hepatol.* **20**, 597–614 (2023). https://doi.org/10.1038/s41575-023-00784-1.
5. Li, M. *et al.* DISCO: a database of Deeply Integrated human Single-Cell Omics data. *Nucleic Acids Res.* **50**, D596–D602 (2022). https://doi.org/10.1093/nar/gkab1020.
6. Hemberg, M. *et al.* Insights, opportunities, and challenges provided by large cell atlases. *Genome Biol.* **26**, 358 (2025). https://doi.org/10.1186/s13059-025-03771-8.
7. Börner, K. *et al.* Human BioMolecular Atlas Program (HuBMAP): 3D Human Reference Atlas construction and usage. *Nat. Methods* 1–16 (2025). https://doi.org/10.1038/s41592-024-02563-5.
8. Weber, G. M., Ju, Y. & Börner, K. Considerations for Using the Vasculature as a Coordinate System to Map All the Cells in the Human Body. *Front. Cardiovasc. Med.* **7**, (2020). https://doi.org/10.3389/fcvm.2020.00029.
9. Ghose, S. *et al.* 3D reconstruction of skin and spatial mapping of immune cell density, vascular distance and effects of sun exposure and aging. *Commun. Biol.* **6**, 718 (2023). https://doi.org/10.1038/s42003-023-04991-z.
10. Jain, Y. *et al.* Exploring endothelial cell environments across organs in spatially resolved omics data. 2025.09.23.678129 Preprint at https://www.biorxiv.org/content/10.1101/2025.09.23.678129v1 (2025). https://doi.org/10.1101/2025.09.23.678129.
11. Black, S. *et al.* CODEX multiplexed tissue imaging with DNA-conjugated antibodies. *Nat. Protoc.* **16**, 3802–3835 (2021). https://doi.org/10.1038/s41596-021-00556-8.
12. He, S. *et al.* High-plex imaging of RNA and proteins at subcellular resolution in fixed tissue by spatial molecular imaging. *Nat. Biotechnol.* **40**, 1794–1806 (2022). https://doi.org/10.1038/s41587-022-01483-z.
13. Hu, J. *et al.* Benchmarking single cell transcriptome matching methods for incremental growth of reference atlases. 2025.04.10.648034 Preprint at https://www.biorxiv.org/content/10.1101/2025.04.10.648034v1 (2025). https://doi.org/10.1101/2025.04.10.648034.
14. Zhang, Y., Aevermann, B., Gala, R. & Scheuermann, R. H. Cell type matching in single-cell RNA-sequencing data using FR-Match. *Sci. Rep.* **12**, 9996 (2022). https://doi.org/10.1038/s41598-022-14192-z.
15. Liu, A. *et al.* Discovery of optimal cell type classification marker genes from single cell RNA sequencing data. *BMC Methods* **1**, 15 (2024). https://doi.org/10.1186/s44330-024-00015-2.
16. Beckett, D., Berners-Lee, T., Prud'hommeaux, E. & Carothers, G. RDF 1.2 Turtle. *RDF 1.2 Turtle* (2025). https://www.w3.org/TR/rdf12-turtle/.
17. Cyberinfrastructure for Network Science Center. Input Data for HRApop v1.0. (2025). https://app.globus.org/file-manager?origin_id=af603d86-eab9-4eec-bb1d-9d26556741bb&origin_path=%2Ff53d60b5994333777a446dd7ad3b0304%2Fextras%2F.
18. Bolin, D. *et al.* Download and Cell Type Annotation (DCTA) Workflow for HRApop v1.0. (2025). https://doi.org/10.5281/zenodo.17368990.
19. Cyberinfrastructure for Network Science Center. Release Download and Cell Type Annotation (DCTA) Workflow for HRApop v1.0 · hubmapconsortium/hra-workflows-runner. *GitHub* (2025). https://github.com/hubmapconsortium/hra-workflows-runner/releases/tag/1.0.
20. Cyberinfrastructure for Network Science Center. hra-workflows-runner:Active Repository for DCTA Workflow. (2025). https://github.com/hubmapconsortium/hra-workflows-runner.

21. Bolin, D. *et al.* HRApop v1.0 CTann Tool Containers. (2025). https://doi.org/10.5281/zenodo.17368954.
22. Cyberinfrastructure for Network Science Center. Release HRApop v1.0 CTann Tool Containers · hubmapconsortium/hra-workflows. *GitHub* (2025). https://github.com/hubmapconsortium/hra-workflows/releases/tag/1.0.
23. Cyberinfrastructure for Network Science Center. hra-workflows: Active Repository for CTann Tool Containers. (2025). https://github.com/hubmapconsortium/hra-workflows.
24. Cyberinfrastructure for Network Science Center. hra-pop/input-data/v1.0 at main · x-atlas-consortia/hra-pop. *GitHub* (2025). https://github.com/x-atlas-consortia/hra-pop/tree/main/input-data/v1.0.
25. Cyberinfrastructure for Network Science Center. hra-pop: Active Repository for RUI2CTpop Workflow. (2025). https://github.com/x-atlas-consortia/hra-pop.
26. Cyberinfrastructure for Network Science Center. hra-pop/scripts at main · x-atlas-consortia/hra-pop. *GitHub* (2025). https://github.com/x-atlas-consortia/hra-pop/tree/main/scripts.
27. Herr II, B. W. & Bueckle, A. hra-pop/output-data/v1.0/reports at main · x-atlas-consortia/hra-pop. *GitHub* (2025). https://github.com/x-atlas-consortia/hra-pop/tree/main/output-data/v1.0/reports.
28. Herr II, B. W., Bueckle, A. & Bolin, D. HRApop v1.0 RUI2CTpop Workflow. (2025). https://doi.org/10.5281/zenodo.17407573.
29. Cyberinfrastructure for Network Science Center. Release RUI to Compile CTpop (RUI2CTpop) Workflow for HRApop v1.0 · x-atlas-consortia/hra-pop. *GitHub* (2025). https://github.com/x-atlas-consortia/hra-pop/releases/tag/1.0.
30. Cyberinfrastructure for Network Science Center. hra-pop/output-data/v1.0/reports/universe-ad-hoc/dataset-info.csv at main · x-atlas-consortia/hra-pop. *GitHub* (2025). https://github.com/x-atlas-consortia/hra-pop/blob/main/output-data/v1.0/reports/universe-ad-hoc/dataset-info.csv.
31. Cyberinfrastructure for Network Science Center. hra-pop/output-data/v1.0/reports/universe-ad-hoc/unmapped-cell-ids.csv at main · x-atlas-consortia/hra-pop. *GitHub* (2025). https://github.com/x-atlas-consortia/hra-pop/blob/main/output-data/v1.0/reports/universe-ad-hoc/unmapped-cell-ids.csv.
32. Cyberinfrastructure for Network Science Center. hra-pop/output-data/v1.0/reports/atlas-ad-hoc/unmapped-cell-ids.csv at main · x-atlas-consortia/hra-pop. *GitHub* (2025). https://github.com/x-atlas-consortia/hra-pop/blob/main/output-data/v1.0/reports/atlas-ad-hoc/unmapped-cell-ids.csv.
33. Cyberinfrastructure for Network Science Center. hra-node-dist-vis/docs/datasets.json at main · cns-iu/hra-node-dist-vis. *GitHub* (2025). https://github.com/cns-iu/hra-node-dist-vis/blob/main/docs/datasets.json.
34. Cyberinfrastructure for Network Science Center. hra-pop/input-data/v1.0/sc-proteomics-cell-summaries.jsonld at main · x-atlas-consortia/hra-pop. *GitHub* (2025). https://github.com/x-atlas-consortia/hra-pop/blob/main/input-data/v1.0/sc-proteomics-cell-summaries.jsonld.
35. Qaurooni, D., Herr II, B. W., Wright, D. & Bueckle, A. HRA Registrations: Manually curated HRA Dataset Graphs. (2025). https://github.com/hubmapconsortium/hra-registrations.
36. Bidanta, S. *et al.* Functional tissue units in the Human Reference Atlas. *Nat. Commun.* **16**, 1526 (2025). https://doi.org/10.1038/s41467-024-54591-6.
37. Bueckle, A. *et al.* The HRA Organ Gallery affords immersive superpowers for building and exploring the Human Reference Atlas with virtual reality. *Front. Bioinforma.* **3**, (2023). https://doi.org/10.3389/fbinf.2023.1162723.

38. Cyberinfrastructure for Network Science Center. HRA Organ Gallery on Horizon Store. *Oculus* (2024). https://www.meta.com/experiences/quest/5696814507101529/.
39. Cyberinfrastructure for Network Science Center. hra-workflows/containers at main · hubmapconsortium/hra-workflows. *GitHub* (2025). https://github.com/hubmapconsortium/hra-workflows/tree/main/containers.
40. Cyberinfrastructure for Network Science Center. hra-pop/output-data/v1.0 at main · x-atlas-consortia/hra-pop. *GitHub* (2025). https://github.com/x-atlas-consortia/hra-pop/tree/main/output-data/v1.0.
